# Genome-wide association analysis of plant architecture traits using doubled haploid lines derived from different cycles of the Iowa Stiff Stalk Synthetic maize population

**DOI:** 10.3389/fpls.2023.1294507

**Published:** 2023-12-04

**Authors:** Alejandro Ledesma, Alice Silva Santana, Fernando Augusto Sales Ribeiro, Fernando S. Aguilar, Jode Edwards, Ursula Frei, Thomas Lübberstedt

**Affiliations:** ^1^ National Institute of Forestry, Crop and Livestock Research, Tepatitlán, Jalisco, Mexico; ^2^ Department of Agronomy, Federal University of Viçosa, Viçosa, Minas Gerais, Brazil; ^3^ Department of Agronomy, Federal University of Lavras, Lavras, Minas Gerais, Brazil; ^4^ Colombian Sugarcane Research Center (Cenicana), Cali, Cauca Valley, Colombia; ^5^ U.S. Department of Agriculture, Agricultural Research Service, Ames, IA, United States; ^6^ Department of Agronomy, Iowa State University, Ames, IA, United States

**Keywords:** candidate gene, quantitative trait locus, diversity, genetic resources, *Zea mays*

## Abstract

Selection in the Iowa Stiff Stalk Synthetic (BSSS) maize population for high yield, grain moisture, and root and stalk lodging has indirectly modified plant architecture traits that are important for adaptation to high plant density. In this study, we developed doubled haploid (DH) lines from the BSSS maize population in the earliest cycle of recurrent selection (BSSS), cycle 17 of reciprocal recurrent selection, [BSSS(R)17] and the cross between the two cycles [BSSS/BSSS(R)C17]. We aimed to determine the phenotypic variation and changes in agronomic traits that have occurred through the recurrent selection program in this population and to identify genes or regions in the genome associated with the plant architecture changes observed in the different cycles of selection. We conducted a *per se* evaluation of DH lines focusing on high heritability traits important for adaptation to high planting density and grain yield. Trends for reducing flowering time, anthesis-silking interval, ear height, and the number of primary tassel branches in BSSS(R)17 DH lines compared to BSSS and BSSS/BSSS(R)C17 DH lines were observed. Additionally, the BSSS(R)C17 DH lines showed more upright flag leaf angles. Using the entire panel of DH lines increased the number of SNP markers identified within candidate genes associated with plant architecture traits. The genomic regions identified for plant architecture traits in this study may help to elucidate the genetic basis of these traits and facilitate future work about marker-assisted selection or map-based cloning in maize breeding programs.

## 1 Introduction

Genetic variability is essential in plant breeding programs. Plant breeders primarily focus on short-term breeding goals, because of the need to deliver new varieties. This may result in a narrow genetic base of maize elite germplasm (Andorf et al., 2019) and could lead to a yield plateau, increased vulnerability to pests and make it difficult to meet new market demands (Pollak, 2003). Assessment of the genetic variability that exists in available germplasm is fundamental for crop improvement. Genetic improvement of important agronomic traits while maintaining genetic variability long-term is desirable in maize breeding programs (Hallauer and Darrah, 1985). In this context, recurrent selection procedures in maize have proven to be effective to increase the frequency of superior lines for grain yield and other agronomic traits while maintaining genetic variability (Hallauer and Darrah, 1985; Ordas et al., 2012; Fritsche-Neto et al., 2023). Recurrent selection is the systematic selection of desirable individuals from a population followed by the selected individuals’ recombination to form the next selection cycle. It was suggested by Jenkins (1940) as a method of intrapopulation improvement and later described for population improvement using a tester (Hull, 1945). The most significant advantage of this method is the increase in the population’s mean performance for one or more traits by increasing the frequency of favorable alleles while maintaining genetic variability for continued genetic improvement. Genetic variability will be preserved if an adequate number of lines is intermated for the next selection cycle.

The Iowa Stiff Stalk Synthetic (BSSS) maize population (Sprague, 1946) has undergone recurrent selection since 1939. This population was developed by intermating 16 inbred lines selected by various maize breeders for superior stalk quality. Of these progenitors, 10 were derived from multiple strains of the Reid Yellow Dent open-pollinated population, 4 had miscellaneous origins, and the genetic background of 2 is unknown (Sprague, 1946). Two recurrent selection programs were initiated in BSSS, a half-sib program with the double cross hybrid IA13 used as a tester and a reciprocal program with the Iowa Corn Borer Synthetic Number One (BSCB1) (Penny and Eberhart, 1971; Eberhart et al., 1973; Martin and Hallauer, 1980; Smith, 1983; Helms et al., 1989; Keeratinijakal and Lamkey, 1993). Two additional programs were initiated using the population generated by seven cycles of half-sib selection (Lamkey, 1992; Edwards 2010). In all four programs selection was carried out for increased grain yield, low grain moisture at harvest, and decreased root and stalk lodging. Several important inbred lines have been developed from the BSSS population (B14, B37, B73, and B84). They have made significant contributions to the maize industry in the US, especially B73, one of the most successful maize inbred lines developed in the public sector and benefited industry and farmers substantially (Coffman et al., 2019).

Agronomic and plant architecture changes have been reported for different selection cycles in the BSSS maize population. These changes involve modifications in traits such as plant height, anthesis-silking interval, leaf angle and number of tassel branches (Brekke et al., 2011; Edwards, 2011). Changes in plant architecture traits over continuous selection cycles, driven by testing under higher population densities have increased throughout the hybrid era (Brekke et al., 2011). In response to increasing plant densities over time, genotypes from later cycles of recurrent selection should have more upright leaves, reduced anthesis-silking interval, and fewer tassel branches.

Genome-wide association studies (GWAS) are a useful tool for analyzing allelic diversity to identify superior alleles and dissect the genetic architecture, which furthers genetic improvement in crops. The increasing application of association mapping is due to the rapid development of sequencing and DNA marker techniques, which resulted in cost-effective high-throughput genotyping technologies. Genomic regions and candidate genes conferring adaptation to high plant density identified by GWAS could help to speed up genetic resource utilization. Identifying genomic regions associated with plant architecture changes may help to unlock genetic resources not adapted to high plant densities, either by selecting such regions in genetic resource populations like early cycles of recurrent selection programs or after introducing them into respective materials (Zhao et al., 2019).

In this study, we phenotypically characterized DH lines developed from the unselected base population, BSSS, the 17^th^ cycle of reciprocal recurrent selection BSSS(R)C17, and the cross between them BSSS/BSSS(R)C17. The purpose of this study was to i) investigate changes in phenotypic diversity for plant architecture traits among DH lines developed from the earliest and the most advanced selection cycle, ii) identify DH lines with both significant C0 background and modern plant architecture traits conferring adaptation to high plant density that could be used as genetic resources, iii) evaluate how to best use DH lines for GWAS from the two subpopulations BSSS and BSSS(R)C17 and the cross between them, to identify regions affecting plant architecture traits, and iv) determine the inheritance of those regions, in particular, whether major genes are involved that may help to accelerate recurrent selection cycles to adapt any germplasm to modern plant types.

## 2 Materials and methods

### 2.1 Breeding populations

Two synthetic populations BSSS, BSSS(R)C17, and the cross between them, BSSS/BSSS(R)C17, representing different stages of cycle advancement in the recurrent selection program of the Iowa Stiff Stalk Synthetic maize population, BSSS, were used to develop DH lines. The synthetic BSSS corresponds to the unselected base population (C0) formed by intermating 16 inbred lines selected for above average stalk quality in 1934 (Sprague, 1946). The C0 seed used came from subsequent cycles of seed multiplication in C0 for maintenance over time. The BSSS(R)C17 population corresponds to the most advanced cycle (C17) available when research was initiated to study crosses between the unselected base population and an advanced cycle. The cross BSSS/BSSS(R)C17 was created by intermating plants from BSSS and BSSS(R)C17.

### 2.2 Doubled haploid line development

Random samples (~1400 plants) from BSSS, BSSS(R)C17, and BSSS/BSSS(R)C17 were pollinated with BHI301, a maternal haploid inducer (Almeida et al., 2020), in an isolation field to generate the haploid seed. Seeds produced from these plants expressing the *R-nj* marker gene in the endosperm but not in the embryo were classified as haploid. The haploid seed was then germinated in plug trays in a greenhouse at the Department of Agronomy, Iowa State University (ISU). Once seedlings developed 2-3 leaves, a colchicine treatment was applied following the DH Facility protocol at ISU (Vanous et al., 2017). Two days after the colchicine treatment, haploid seedlings were transplanted in the field at the Agricultural Engineering and Agronomy Research Farm, Boone, Iowa. Putative DH0 plants shedding pollen were self-pollinated to produce DH1 generation seed. Seed multiplication was performed during subsequent growing seasons, and lines were screened for uniformity and discarded if segregating. In total, 135, 194 and 187 DH lines from BSSS (C0_DHL), BSSS(R)17 (C17_ DHL) and BSSS/BSSS(R)C17 (C0/C17_DHL), respectively, were obtained.

### 2.3 Experimental design and phenotypic data collection

The 516 DH lines plus 16 progenitors of the BSSS population [A3G-3-3-1-3, CI 540, Fe (Parent of F1B1), I-159, IL12E, B2 (Parent of F1B1), Oh 3167B, Os 420, Tr 9-1-1-6, WD 456, I224, LE23, Ind. 461, Hy, AH83, CI 187-2] and the inbred line B73 were planted during summer 2019 at three locations: Plant Introduction Station (PI) in Ames, IA, Johnson Farm near Kelly, IA, and Burkey at Agronomy Farm near Boone, IA. The experiment was planted in each location using a modified split plot design with two replications, where the DH lines for populations BSSS, BSSS(R)C17, and BSSS/BSSS(R)C17 constituted the whole plot treatment factor and the DH lines within each population the subplot treatment factor. This design differs from a classical split-plot because the subplot factor (DH lines) was nested within the whole-plot factor, population. Progenitors were included as subplot treatments within BSSS whole plots. Inbred line B73 was used as a check and replicated 14 times within each replicate resulting in 546 experimental units per replication (516 DH lines, 16 progenitors, and 14 replicates of B73) The subplot experimental unit consisted of a single row plot, 3.8 m long with 15 plants with 0.76 m between rows. The whole-plot factor experimental unit was a block containing 39 subplots arranged side by side. Each replication, containing 546 subplots, was divided into three whole plots, which were then separated into 4, 5, and 5 blocks for C0_DHL, C17_DHL, and C0C17_DHL, respectively. Each whole-plot block was randomly assigned to a range in the field.

Phenotypic data were collected on a plot basis for male flowering, female flowering, plant height, ear height, flag leaf angle, tassel length, and the number of primary tassel branches. Male flowering and female flowering were recorded as the date when 50% of the plants in the row were shedding pollen and had visible silks, respectively. Plants were recorded as shedding pollen when a single anther could be seen, and plants were recorded as silking, when one or more silks were visible. Anthesis-silking interval was calculated as the difference in days between male flowering and female flowering. Plant and ear height were recorded two weeks after pollination: plant height was the height (cm) from the soil surface to the flag leaf collar and ear height was the height (cm) from the soil surface to the stalk node at which the uppermost ear has emerged. The flag leaf angle was recorded using a protractor. The protractor was placed against the portion of stalk beneath the flag leaf. The protractor was held underneath the flag leaf’s midrib to record the flag leaf angle at the point of attachment to the stalk. Tassel length was measured two weeks after pollination as the length (cm) between the flag leaf node and the top of the tassel. The number of primary tassel branches was recorded simultaneously as tassel length by counting the number of primary tassel branches that branch directly off the main branch.

### 2.4 Statistical data analysis

Data were analyzed with the following linear model:


$$Y_{ijklmn}=\;µ\;+\;E_{i}+\;R{\left(E\right)}_{li}+\;G_{j}+\;GE_{ij}+\;D{\left(G\right)}_{jk}+\;ED{\left(G\right)}_{ijk}+\;P{\left(ER\right)}_{mil}+\;A{\left(ER\right)}_{nil}+\epsilon_{ijklmn}$$

where: Y_ijklm_ is the response in the environment *i*, group *j*, DH line *k*, replicate block *l*, pass *m* (i.e., field rows), range *n* (i.e., field columns); µ is the overall mean; E*_i_* is the effect of environment *i*; R(E)*_li_* is the effect of replicate block *l* within environment *i*; G*_j_* is the effect of the group of DH line *j*; GE*_ij_* is the effect of the interaction between group *j* and environment *i*; D(G)*_jk_* is the effect of the DH line *k* within the group *j*; ED(G)*_ijk_* is the effect of the interaction between environment *i* and DH line *k* within the group of DH line *j*; P(ER)*_mil_* is the effect of the pass *m* within the environment *i* and replication *l*; A(ER)_nil_ is the effect of the range *n* within the environment *i* and replication *l* and ϵ*_ijklm_* is the effect of the residual error of the range *n*, pass *m*, block *l*, individual DH line *k*, group of DH line *j* and environment *i*. The effects of the environment, replicate block within environment, group of DH lines were considered fixed effects. All other effects were considered random. All phenotypic data analyses were conducted using the MIXED procedure of SAS 9.4 software (SAS Institute, Cary, NC). After fitting the full linear model to all traits, data were checked for outliers by computing the probability of studentized residuals using the t-distribution and adjusted with a Bonferroni correction for the number of residuals. Observations were considered outliers if the Bonferroni corrected P-value on the residuals were below 0.02. Then, a model containing all fixed effects but with different combinations of the random effects and homogeneity/heterogeneity in the residual variance across environments was tested for each trait.

Based on the smallest Bayesian Information Criteria (BIC; Schwarz, 1978), we decided which random effects to retain in the model. A final model was identified as having the best fit for each trait. The model with the smallest BIC value is shown in supplemental materials (**Supplementary Table 1**). Variance components were estimated by REML (Patterson and Thompson, 1971), and likelihood ratio tests were performed to verify the significance of them. Overall means of the DH line groups were compared using Tukey’s honest significant difference (HSD) procedure. **Supplementary Table 2** shows BLUP values for 132, 185 and 170 DH lines from C0_DHL, C17_DHL and C0/C17_DHL groups, respectively. This information should be used to identify DH lines with both significant C0 background and modern plant architecture traits conferring adaptation to high plant density.

Repeatability was calculated with the formula:


$$\text{Repeatability }=\;\frac{{\hat{\sigma }}_{D\left(G\right)}^{2}}{{\hat{\sigma }}_{D\left(G\right)}^{2}+\frac{{\hat{\sigma }}_{ED\left(G\right)}^{2}}{e}+\frac{{\hat{\sigma }}_{\text{ϵ}}^{2}}{re}}$$

where ${\hat{\sigma }}_{D\left(G\right)}^{2}$ corresponds to the variance estimate due to the DH line within group effect, ${\hat{\sigma }}_{ED\left(G\right)}^{2}$ is the variance estimate due to the interaction between environment and DH line, ${\hat{\sigma }}_{\text{ϵ}}^{2}$ is the residual variance estimate and *r* and *e* are the number of replications and environments, respectively (Carena et al., 2010). The Pearson correlation coefficients between BLUPs from D(G)*_jk_* effect were calculated using the R software (R Core Team, 2021).

### 2.5 Genotyping and quality control

Genomic DNA was extracted from each DH line seedling established in the greenhouse at the Department of Agronomy, ISU. Leaf tissue samples from three plants per DH line were collected at the 3-4 leaf developmental stage, and the DNA extraction was done using the standard CIMMYT laboratory protocol (CIMMYT, 2005). Genotyping was carried out using the Diversity Arrays Technology sequencing (DArT-seq) method (Kilian et al., 2012) provided by the Genetic Analysis Service for Agriculture (SAGA) at CIMMYT. DArT-seq is a high-throughput, robust, reproducible, and cost-effective marker system based on genome complexity reduction using a combination of restriction enzymes, followed by hybridization to microarrays to simultaneously assay hundreds to thousands of markers across the genome (Sansaloni et al., 2011).

A total of 51,418 SNP markers were generated, but only 32,929 SNP markers were successfully called within the B73 RefGen_v5. The 32,929 SNP markers were filtered according to the following criteria: 1) Minimum call rate, 2) Minor Allele Frequency (MAF), 3) duplicated and monomorphic markers, and 4) heterozygosity. We used a threshold of ≥ 50% to remove poorly genotyped SNP markers, for which information was missing for more than half of the lines. SNP markers with MAF ≤ 1% were excluded. Duplicated and monomorphic SNP markers were removed using conditional formatting in Excel. Finally, genotypes with significant heterozygosity (not expected in DH or inbred lines) were excluded. After filtering and quality control, 13,846 SNP markers remained. In total, 29 DH lines (3 in C0_DHL, 9 in C17_DHL, and 17 in C0/C17_DHL) were discarded from the GWAS analysis due to obvious phenotypic segregation observed in field trials or missing genotypic or phenotypic data.

The software TASSEL v.5.2.70 (Bradbury et al., 2007) was used for the imputation of missing data using the LDkNNi (linkage disequilibrium k-nearest neighbors imputation) method (Money et al., 2016). LDkNNi process considers the linkage disequilibrium (LD) between SNPs when choosing the nearest neighbors. It exploits the fact that markers useful for imputation are often not physically close to the missing genotype rather distributed throughout the genome (Money et al., 2016).

### 2.6 Linkage disequilibrium and population structure

The average LD decay between SNP markers for each chromosome was determined in each group of DH lines using the squared Pearson correlation coefficient (r^2^) between alleles at two loci for all possible combinations of alleles, and then weighting them according to the allele frequency. P-values were determined by a two-sided Fishers Exact test (Bradbury et al., 2007). The option “Full Matrix LD” on TASSEL v.5.2.70 was used to calculate LD for every combination of sites in the alignment (Bradbury et al., 2007). The resulting data were imported into R (R Core Team, 2021) to create LD decay plots and fit a smooth line using Hill and Weir expectations of r^2^ between adjacent sites (Hill and Weir, 1988).

The selected 487 DH lines were known to belong to the three subpopulations BSSS, BSSS(R)C17, and BSSS/BSSS(R)C17. A principal component analysis (PCA) was conducted for all DH lines using the software GAPIT v.3 (Lipka et al., 2012). The principal components, plotted in a two-dimensional plot using discriminant analysis of principal components (DAPC), correctly identified a clear grouping of the DH lines into the three groups (C0_DHL, C17_DHL and C0/C17_DHL). The first two principal components explained 14.3% of the total SNP variation in the entire panel. Also, the C0/C17_DH lines group was scattered over a broader range, similar to the C0_DHL group. PCA results and molecular characterization of the DH lines within and among the cycles of selection are presented in Ledesma et al. (2023). The incorporation of population structure through PCA as a covariate in the fixed effect model increases the power to detect associations, and it has the advantage of eliminating false positives due to non-genetic effects associated with the population structure.

### 2.7 Genome-wide association studies

For GWAS analyses, we used four phenotypic traits that are known to be associated with adaptation to high plant density: male and female flowering, flag leaf angle, and the number of primary tassel branches. GWAS analysis was performed for each subpopulation individually (C0_DHL, C17_DHL, and C0/C17_DHL) and for the entire panel (487 DH lines) in order to determine how to best use DH lines for GWAS. The software package GAPIT (Lipka et al., 2012) was used for GWAS analysis. The fixed and random model circulating probability unification (FarmCPU) method was implemented in GAPIT. FarmCPU includes PCA results as a covariate, kinship as an additional covariate to account for the relatedness among individuals (VanRaden, 2008), and additional algorithms that aid in solving the confounding problem between testing markers and covariates (Liu et al., 2016).

The P-values from each respective SNP were adjusted using False Discovery Rate (FDR) according to the Benjamini and Hochberg method (Benjamini and Hochberg, 1995). This statistic is also known as q-value and represents the estimated FDR if the associated P-value is used to declare significance. The default significant threshold value implemented in GAPIT was set at FDR < 0.05. We used the uniform Bonferroni-corrected threshold of α = 0.05 for the significance level. Therefore, the suggested P-value was computed with α/n (n = 13,846, total markers used), and we obtained a P-value threshold of 3.61×10^-6^ for GWAS. Manhattan plots were used to visualize the significance of SNPs by chromosome location across the whole genome for each trait. Allele frequencies within population were estimated for each significant SNP by using the *popgen* function from snpReady R package (Granato et al., 2018).

### 2.8 Candidate gene mining

The available maize genome sequence (B73; RefGen_v5) was used as the reference genome for candidate gene identification. Genes were considered as candidates if a significantly associated SNP marker with phenotypic variance explained (PVE) higher than 5% was located within the range of LD decay observed for each chromosome (upstream and downstream). Candidate genes were identified using the Ensembl Biomart tool (Kinsella et al., 2011) and checked according to the SNP marker’s physical position in the MaizeGDB molecular marker database (http://www.maizegdb.org; Portwood et al., 2019). Functional annotations of candidate genes were predicted in NCBI (http://www.ncbi.nlm.nih.gov/gene) and were also compared to previously published candidate genes.

## 3 Results

### 3.1 Phenotypic data analyses

Descriptive statistical analysis confirmed trait variability in the different groups of DH lines (**Table 1**). Phenotypic differences (*P* ≤ 0.05) for all traits, except plant height, were found among groups of DH lines. DH lines within the C0_DHL group had the highest mean values for flowering time, ear height, flag leaf angle, tassel length and the number of primary tassel branches and were found to be different (*P* ≤ 0.05) between the C17_DHL and C0/C17_DHL groups. On the other hand, DH lines within C17 group had the lowest values for these traits (**Table 1**). The C17_DHL group had the smallest anthesis-silking interval (0.1), meaning that plants showed silks and pollen shed almost simultaneously. Variance components due to DH lines within group effect were significant (*P*< 0.05) by the likelihood ratio test for all traits. Repeatabilities calculated for the complete set of DH lines across the three locations were found to be high across all traits. They ranged from 0.82 to 0.94 (**Table 1**). The correlation between the BLUPs were explored to determine relationships among evaluated traits (**Table 2**). The closest positive correlation (r = 0.88) was observed between male flowering and female flowering (*P* ≤ 0.001). Plant and ear height were significantly (*P* ≤ 0.001) and positively correlated (r = 0.76). They were also significantly and positively correlated with almost all other traits, except for the number of primary tassel branches and anthesis–silking interval.

**Table 1:** Statistics of flowering and plant architecture traits in different groups of DH lines derived from the BSSS maize population.

Trait	Group^a^	Mean	${\hat{\sigma }}_{D\left(G\right)}^{2}$	Repeatability
Male flowering (days)	C0_DHL	67.4 a	6.8*	0.94
	C17_DHL	62.7 c	4.2*	
	C0/C17_DHL	65.5 b	5.2*	
Female flowering (days)	C0_DHL	69.1 a	8.5*	0.94
	C17_DHL	62.7 c	4.7*	
	C0/C17_DHL	66.4 b	7.7*	
Anthesis-silking interval (days)	C0_DHL	-1.7 a	1.6*	0.82
	C17_DHL	0.1 c	0.8*	
	C0/C17_DHL	-0.9 b	1.6*	
Plant height (cm)	C0_DHL	169.4 a	274.2*	0.93
	C17_DHL	170.5 a	194.4*	
	C0/C17_DHL	172.6 a	246.3*	
Ear height (cm)	C0_DHL	83.5 a	216.5*	0.92
	C17_DHL	68.5 c	122.4*	
	C0/C17_DHL	79.6 b	212.2*	
Flag leaf angle (Degrees from vertical)	C0_DHL	42.2 a	158.2*	0.89
	C17_DHL	13.8 c	48.1*	
	C0/C17_DHL	30.6 b	146.8*	
Tassel length (cm)	C0_DHL	42.1 a	16.1*	0.90
	C17_DHL	36.9 c	14.7*	
	C0/C17_DHL	38.9 b	21.1*	
Primary tassel branches (number)	C0_DHL	15.4 a	1.5*	0.94
	C17_DHL	7.3 c	0.4*	
	C0/C17_DHL	10.4 b	0.8*	

a Group, C0_DHL corresponds to the 132 derived DH lines from cycle 0, C0/C17_DHL corresponds to the 170 derived DH lines from C0/C17, and C17 corresponds to the 187 derived DH lines from cycle 17. Mean values were estimated from trait BLUPs of n lines within each group; ${\hat{\sigma }}_{D\left(G\right)}^{2}$ = variance estimate due to DH lines within group effect; * significant at 0.01 by the likelihood ratio test.

Means with the same letter in column are not statistically different at the 0.05 level of probability using Tukey’s HSD comparison.

**Table 2:** Pearson correlation coefficients (r) between BLUPs for flowering and plant architecture traits of DH lines developed from the BSSS maize population.

	MAFL	FEFL	ASI	PLHE	EAHE	FLA	TALE	NPTB
MAFL	1							
FEFL	0.88**	1						
ASI	-0.02	-0.48	1					
PLHE	0.20**	0.14**	0.06	1				
EAHE	0.36**	0.27**	0.10*	0.76**	1			
FLA	-0.04	-0.05	0.02	0.10*	0.15*	1		
TALE	-0.05	0.01*	-0.11	0.24**	0.13*	-0.07	1	
NPTB	0.02	0.08*	-0.12	-0.02	0.07	0.10*	-0.04	1

** Significant at P ≤ 0.001, * Significant at P ≤ 0.05.

MAFL, male flowering; FEFL, female flowering; ASI, anthesis–silking interval; PLHE, plant height; EAHE, ear height; FLA, flag leaf angle; TALE, tassel length; NPTB, number of primary tassel branches.

### 3.2 Linkage disequilibrium

**Figure 1:**
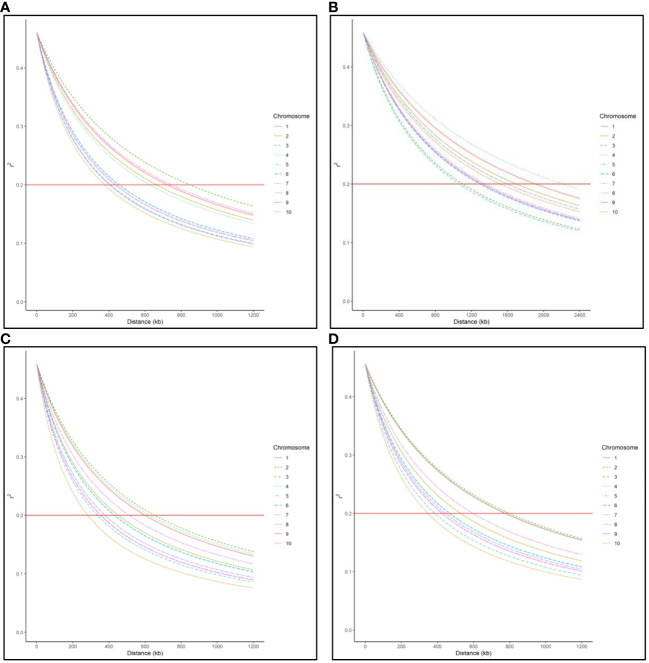
Genome-wide LD decay distance in **(A)** C0_DHL group, **(B)** C17_DHL group, **(C)** C0/C17_DHL group, and **(D)** Entire panel of 487 DH lines.

LD decay varied across the ten chromosomes and different regions within chromosomes (**Figure 1**). The C17_DHL group showed the largest LD decay distance ranging from 1,067 to 2,218 kb on chromosomes 5 and 4, respectively (**Table 3**). In contrast, the C0/C17_DHL group displayed the smallest LD decay distance (from 284 kb on chromosome 10 to 653 kb on chromosome 3). For C0_DHL, the LD decay ranged from 377 to 848 kb on chromosomes 10 and 3, respectively. The genome-wide LD decay distance was 569 kb, 1,509 kb and 463 kb for the C0_DHL, C17_DHL and C0/C17_DHL groups, respectively (**Table 3**). The genome-wide LD decay distance over all ten chromosomes in the entire panel of DH line panel was equal to 555 kb.

**Table 3:** Linkage disequilibrium decay distance (kb) per chromosome in the different groups of DH lines and the entire panel.

Chromosome	C0_DHL	C17_DHL	C0/C17_DHL	Entire panel
1	724	1,911	600	774
2	663	1,698	452	529
3	848	1,104	653	799
4	627	2,218	625	781
5	408	1,067	336	386
6	456	1,298	431	467
7	404	1,597	379	445
8	748	1,352	512	597
9	436	1,320	355	426
10	377	1,523	284	348
Genome-wide LD	569	1,509	463	555

### 3.3 Genome-wide association studies

**Figure 2:**
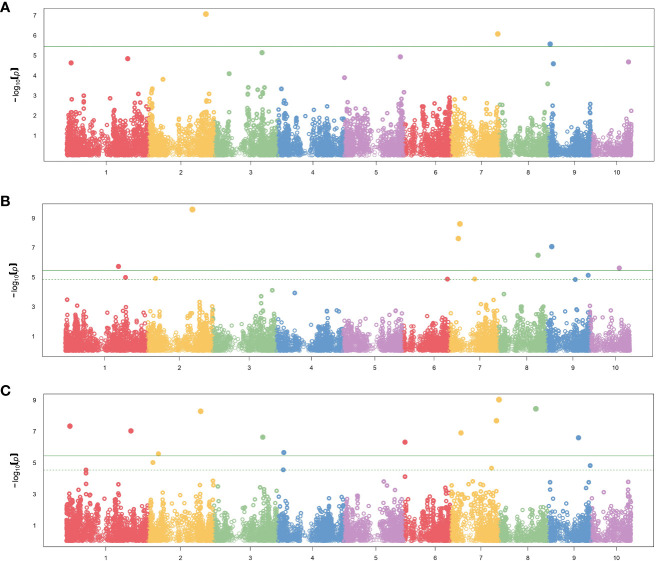
Manhattan plot results showing significant SNP markers associated with **(A)** female flowering, **(B)** flag leaf angle, **(C)** number of primary tassel branches in the entire panel using FarmCPU method. The X-axis plot represents the genomic position of the SNPs per chromosome. The Y-axis represents the negative logarithm of the P-value obtained from the GWAS model. The dash horizontal line represents the threshold from the FDR, and the solid horizontal line represents the threshold from the Bonferroni correction method.

In total, 26 significant SNP markers were identified by FarmCPU (**Table 4**). A greater number of significant SNPs was found when the entire panel of DH lines (487 DH lines) was combined and used for GWAS with FarmCPU model. Therefore, the associations from the entire panel were considered for further analyses. A total of 22 SNP markers were found significant when using FarmCPU model with the entire panel of DH lines. Among those, two and one SNP presented PVE higher than 5% for flag leaf angle and number of primary tassel branches, respectively (**Figure 2**; **Supplementary Table 3**). No significant SNP was detected for male flowering trait (**Table 4**). By searching for candidate genes up and downstream for those three SNP markers being in LD with the corresponding chromosome based on the B73 RefGen_v5, 19 candidate genes were identified (**Table 5**). Ten candidate genes were identified for flag leaf angle and nine for number of primary tassel branches. We observed that for most significant SNPs, the allele frequencies were lower within C0_DHL, intermediate within C0C17_DHL and highest within C17_DHL population (**Supplementary Table 4**).

**Table 4:** The number of significant SNP markers associated with flowering and plant architecture traits in different groups of DH lines and the entire panel using FarmCPU model.

Population	Phenotypic traits				
	MAFL	FEFL	FLA	NPTB	Total
C0_DHL	0	0	0	0	0
C17_DHL	0	4	0	0	4
C0/C17_DHL	0	0	0	0	0
Entire panel	0	3	7	12	22
Total	0	7	7	12	26

MAFL, male flowering; FEFL, female flowering; FLA, flag leaf angle; NPTB, number of primary tassel branches.

**Table 5:** Candidate genes associated with plant architecture traits in the BSSS DH lines.

Traits	Chr	Gene start (Kbp)	Gene ID MaizeGDB	Gene ID Gramene	Gene name	Annotation
Flag leaf angle	1	199659	Zm00001eb036930	GRMZM2G136453	pap15	purple acid phosphatase15
	1	199726	Zm00001eb036940	GRMZM2G043198	pdh2	pyruvate dehydrogenase2
	1	199884	Zm00001eb036970	GRMZM2G159996	col16	C_2_C_2_-CO-like-transcription factor 16
	1	200045	Zm00001eb036990	GRMZM5G882527	bhlh173	bHLH-transcription factor 173
	1	200445	Zm00001eb037120	GRMZM2G033828	rrb3	retinoblastoma family3
	1	199275	Zm00001eb036880	GRMZM5G872141	sweet11	sugars will eventually be exported transporter11
	1	199317	Zm00001eb036890	GRMZM2G068657	pat4	protein S-acyltransferase4
	1	199461	Zm00001eb036910	GRMZM2G064962	gpx3	glycerophosphodiester phosphodiesterase3
	2	167752	Zm00001eb095620	GRMZM2G161382	cyc11	cyclin11
	2	168274	Zm00001eb095690	GRMZM2G087955	myb139	MYB-transcription factor 139
Number of primary tassel branches	2	194428	Zm00001eb101630	GRMZM2G022162	ca5p12	CCAAT-HAP5-transcription factor 512
	2	194465	Zm00001eb101670	GRMZM2G003992	mlkp3	Maize LINC KASH AtWIP-like3
	2	194543	Zm00001eb101700	GRMZM2G052671	wrky71	WRKY-transcription factor 71
	2	194575	Zm00001eb101720	GRMZM2G350857	abi53	ABI3-VP1-transcription factor 53
	2	194727	Zm00001eb101780	GRMZM2G080439	upl1	ubiquitin-protein ligase1
	2	194795	Zm00001eb101840	GRMZM2G134334	znf13	zinc finger protein13
	2	194921	Zm00001eb101880	GRMZM2G417597	bhlh6	bHLH-transcription factor 6
	2	195046	Zm00001eb101910	GRMZM2G125934	bzip85	bZIP-transcription factor 85
	2	195064	Zm00001eb101920	GRMZM2G126018	sbp23	SBP-transcription factor 23

## 4 Discussion

### 4.1 Plant architecture traits adapting to high plant density

The breeding potential of the BSSS maize population DH lines is reflected by the distribution of the plant architecture traits that have been modified in this population, and these traits are involved in the adaptation to high plant densities (Duncan et al., 1967; Duncan, 1971; Mock and Pearce, 1975; Brekke et al., 2011). C17_DHL group presented the most favourable traits when adapting germplasm to higher plant densities (**Table 1**), such as reduced anthesis-silking interval, more erect leaves, and fewer primary tassel branches. The phenotypic data used in our study showed high values of repeatability, ranging from 0.82 to 0.94. These repeatabilities values agree with other studies (Buckler et al., 2009; Romay et al., 2013; Peiffer et al., 2014; Vanous et al., 2018).

In this study, we found significance differences in the mean of the plant architecture traits among the group of DH lines and a reduction in the variance component estimates from the C17_DHL to the C0_DHL. Reduced genetic variance within the population was expected after 17 cycles of recurrent selection with recombination of a finite number of lines (10 or 20) within each cycle of selection. Flowering time showed a reduction of four days to anthesis and six days to silking from C0_DHL to C17_DHL groups. However, all DH lines flowered within a timeframe expected for the central US Corn Belt. Reduction in flag leaf angle has been reported in hybrids through the selection process and adaptation to high plant density (Duvick, 2005), as we found in this study. C17_DHL group could be a source of favourable alleles that impact more erect flag leaf angles. Additionally, we found a reduction in the number of primary tassel branches from an average of 15 in C0_DHL to 7 in the C17_DHL groups. These results confirmed a reduction in the number of primary tassel branches found by Edwards (2011) in the recurrent selection in the BSSS maize population. Additionally, these results are also in agreement with Brekke et al. (2011), where changes in plant architecture traits such as more upright flag leaf angle and reduction on the number of tassel branches were found as the cycles of selection advanced in the BSSS maize population. Large tassels can intercept enough light to lower photosynthetic rates in the canopy (Duncan et al., 1967), suggesting that smaller tassels may be advantageous for light utilization. However, Duncan et al. (1967) pointed out that this does not necessarily preclude some benefit of improved assimilate allocation with smaller tassels.

Ear height for the C17_DHL group was significantly lower than for C0_DHL, which might be partially due to the inbreeding depression. Plant and ear height are traits of interest when adapting germplasm as they are closely associated with flowering time, lodging resistance, biomass production, and grain yield (Durand et al., 2012; Teng et al., 2013). By reducing height traits during the selection for industrial agriculture, it was observed an increased harvest uniformity, favourably partition carbon and nutrients between grain and non-grain biomass, and enhanced fertilizer, pesticide, and water use efficiency (Khush, 2001). C17_DHL group was altered in important traits for high plant density tolerance compared to the C0_DHL group. In general, the C17_DHL group showed a better performance in plant architecture traits than the C0_DHL group. These differences demonstrate that 17 cycles of recurrent selection have been effective. At the same time, the C0/C17 DHL group showed considerable variation and could be used as a source to develop DH lines and hybrids adapted to high planting densities. Developing DH lines in more advanced cycles of selection improved agronomic traits, such as flowering time, flag leaf angle, and the number of primary tassel branches. If there were few major loci available in early selection cycles, they probably got fixed during the selection process. Therefore, the extraction of DH lines out of the BSSS maize population was effective, as indicated by plant architecture traits that suggested adaptation to high plant density. Some correlations coefficients were significant, indicating that adaptation based on plant architecture traits is a viable option in altering other important adaptation-related traits.

### 4.2 The exploitation of early cycle of BSSS DH lines

A method to exploit maize’s genetic diversity is introducing exotic germplasm and/or using landraces as a source of new alleles. However, several cycles of inbreeding are required. Additionally, inbreeding from landraces results in a high load of recessive alleles, mutations, and deleterious alleles that need to be selected against by conventional breeding methods (Strigens et al., 2013). According to Ledesma et al. (2023), the 17 cycles of reciprocal recurrent selection program have left behind useful genetic variation present in the C0_DHL during the selection process. Thus, to have a sufficient number of lines to be evaluated for testcross performance from exotic germplasm or landraces, it is necessary to start the breeding program with a large number of plants. This laborious effort is the main reason why exotic germplasm and landraces are limited used in modern breeding programs (Goodman, 2005). However, DH technology can enable more effective access to the genetic diversity of landraces and exotic germplasm in a faster way (Strigens et al., 2013; Chaikam et al., 2019). In this context, the C0_DHL group could be a reservoir of genetic diversity that could be untapped using DH technology. Deleterious alleles are expressed in the haploid stage and can be purged through selection. Hence, DH technology is a useful tool to access the genetic diversity present in landraces and to expand the genetic diversity of the elite germplasm (Wilde et al., 2010; Strigens et al., 2013; Böhm et al., 2017; Chaikam et al., 2019).

Developing DH lines from earlier cycles of recurrent selection programs could be an alternative approach to conventional breeding for introduction of diversity into related elite lines. In this study, we developed DH lines from the earlier cycle of the BSSS maize population to explore the phenotypic variation that has been left behind when advancing cycles of recurrent selection. Significant phenotypic variation was observed between the groups of DH lines for all traits evaluated, except for plant height. C17_DHL group presented the most favorable characteristics when adapting germplasm to higher plant densities (i.e., lowest means for flowering time, ear height, flag leaf angle, tassel length and the number of primary tassel branches). However, the genetic variability among the C0_DHL and the C0/C17_DHL allowed the identification of DH lines with desirable plant architecture traits that confer adaptation to high plant density. Some of these DH lines are a promising source of favorable alleles for plant density response. Thus, selected DH lines could be introgressed into current germplasm to improve the adaptation to high plant density. The large genetic distances of the C0_DHL compared to the C17_DHL (Ledesma et al., 2023) demonstrated the potential of the C0_DHL group to broaden the genetic base of the Stiff Stalk (SS) germplasm. However, more studies need to be conducted at the testcross level to know the hybrid combinations’ performance. The use of early selected cycles and DH technology opens new opportunities for exploring genetic diversity in available germplasm.

### 4.3 Linkage disequilibrium and GWAS analysis

LD refers to the nonrandom association of alleles at different loci in a breeding population (Flint-Garcia et al., 2003). It can be estimated using the correlation between SNP markers. The magnitude of LD and its decay with the genetic distance is important to determine the resolution of association mapping because LD’s extent determines the required number of SNP markers and the mapping resolution (Vos et al., 2017). In our entire panel of BSSS DH lines, we found that the LD decayed over 555 kb across the genome at the r^2 = ^0.2 threshold (**Figure 1D**). However, LD decay varied across the ten chromosomes and different genetic regions within chromosomes ranging from 348 kb in chromosome 10 to 799 kb in chromosome 3 (**Figure 1D**). These results agree with Vanous et al. (2018). They investigated a diverse panel consisting of exotic derived DH lines and found that LD decayed over a distance greater than 500 kb for all chromosomes. The LD within the C17_DHL group is quite more extensive than in C0_DHL and C0/C17_DHL. The larger LD decay distance observed in the C17_DHL group may be due to the breeding history of the population (e.g., the occurrence of bottlenecks) and the lower genetic diversity represented by this population. LD decay is more rapid in pools with higher genetic diversity (Romay et al., 2013; Wu et al., 2016). The C17_DH lines came from a population that was gone through 17 cycles of recurrent selection, which have caused some genetic drift, or a small effective population size, resulting in the larger decay distances.

The rapid LD decay, together with high genotypic variances and absence of population structure within populations, enables good resolution association mapping in some germplasm (Strigens et al., 2013). In our study, when we analyzed each group of DH lines (C0_DHL, C17_DHL, and C0/C17 DHL) the number of SNP markers associated was low or absent. However, when we used the entire panel of DH lines, we found 22 SNP markers among all traits. These results could be due to the lower variation within each DH line group or the smaller population size that affects the power to detect associations. Another possible reason for having low power to identify associated SNP markers to plant architecture traits when we performed the analysis by each group of DH lines could be due to the fixation of alleles. In the C17_DHL group, there are major genes affecting plant architecture traits and respective alleles are present at a low frequency in the C0_DHL group.

Alleles were in higher frequency within the population C17_DHL (**Supplementary Table 4**). The intermediate allele frequencies observed within population C0C17_DHL suggests that this population might be the most powerful population for GWAS studies, as those lines segregate for favorable alleles. Most significant SNPs were detected when using the entire panel not only because of its population structure, but mainly because the sample size was higher when using the entire panel. It has been largely discussed that sample size plays an important role in GWAS studies (Ibrahim et al., 2020; Wang et al., 2020; Murphy et al., 2022). Therefore, we believe that an increased sample size of C0C17_DHL could increase its power of SNP detection.

### 4.4 Candidate genes for plant architecture traits adapting to high plant density

Since we found consistent changes in at least four traits that are known to be associated with adaptation to high plant density we focused our discussion on candidate genes for these traits. Trends for reducing male and female flowering time, the number of primary tassel branches, and more upright flag leaf angles in C17_DHL compared with the C0_DHL were identified in our work. These trends have been reported for parental inbred lines of hybrids previously released (Duvick, 2005; Lauer et al., 2012), which could reflect a correlated response of modern breeding germplasm to selection for grain yield under higher plant densities (Edwards, 2011).

Flag leaf angle and number of primary tassel branches presented significant SNP markers with PVE higher than 5% (**Supplementary Table 3**). Thus, we searched potential candidate genes for these two traits. In total, 19 candidate genes were found. Flag leaf angle has experienced changes when advancing cycles in the recurrent selection program. In this study, we found that C17_DHL had a more upright flag leaf angle than the other two groups of DH lines. These results agree with different hybrids studies where a trend toward vertical flag leaf angle had been observed in recent decades. More vertical upper leaves are the desired trait since permit lighter to penetrate the canopy, improving the photosynthetic efficiency and allowing farmers to plant maize at higher densities (Edwards, 2011). In this study, an important region on chromosome 7 with PVE equal to 10.81% was identified. This suggests that the surrounding genomic region might have a strong association on modifying flag leaf angle, which could help to dramatically alter the trait.

Early studies conducted in maize to dissect the genetic basis of leaf angle have identified several quantitative trait loci and genomic regions for leaf angle throughout all the ten maize chromosomes. Dzievit et al. (2019) found 12 QTL on chromosome 1, 2, 3, 4 and 8 affecting leaf angle. Additionally, several genes have been cloned as the outcome of the combined use of quantitative genetics and induced or natural mutants associated with changes in leaf angle in maize (Mantilla-Perez and Fernandez, 2017). The number of primary tassel branches is considered as the principal component of maize tassel inflorescence architecture and is a typical quantitative trait controlled by multiple genes (Chen et al., 2017). Reductions in tassel size and tassel branch number have continuously decreased over time (Duvick, 2005). Previous studies in the BSSS maize population have revealed changes through advancing cycles in the recurrent selection program (Brekke et al., 2011). According to Duncan et al. (1967), tassels could block enough sunlight to reduce photosynthesis by 19%. We identified nine candidate genes controlling the number of primary tassel branches which will be useful for its improvement by molecular breeding and provide a basis for the cloning of the genes. Chen et al. (2017) identified 11 QTL located in chromosomes 2, 3, 5, and 7 demonstrating that tassel branch number variation was mainly caused by alleles with a major effect, minor effect, and slightly modified by epistatic effects.

DH lines developed in this study could be sources of new germplasm for broadening the genetic variation compared to elite germplasm to develop varieties or hybrids adapted to the US corn belt. Thus, individual lines with superior performance for agronomic and morphological traits can be selected and introgressed into elite materials. However, the testcross performance of the DH lines remains to be evaluated to test their yield potential in hybrid combinations. Additionally, in this study, we found that the entire panel of DH lines could be used for association analysis for flowering and plant architecture traits. Instead of using each DH line group individually, the power of detecting associated SNP increased when we used the entire panel of DH lines. Additionally, identifying QTL or regions for plant architecture traits in this study may help to elucidate the genetic basis of these traits and facilitate future work about marker-assisted selection or map-based cloning in maize breeding programs.

## Data availability statement

The datasets presented in this study can be found in online repositories. The names of the repository/repositories and accession number(s) can be found below: https://doi.org/10.25380/iastate.22893878.v1, Iowa State University DataShare, accession 22893878.

## Author contributions

AL: Data curation, Formal analysis, Investigation, Methodology, Project administration, Writing – original draft, Writing – review & editing. AS: Data curation, Formal analysis, Software, Validation, Writing – review & editing. FR: Writing – review & editing. FA: Data curation, Formal analysis, Methodology, Writing – original draft, Writing – review & editing. JE: Conceptualization, Funding acquisition, Methodology, Project administration, Resources, Supervision, Validation, Writing – original draft, Writing – review & editing, Investigation. UF: Data curation, Project administration, Supervision, Validation, Visualization, Writing – original draft, Writing – review & editing. TL: Conceptualization, Funding acquisition, Investigation, Methodology, Project administration, Resources, Supervision, Validation, Visualization, Writing – original draft, Writing – review & editing.
