# Supplementary material for: Genome-wide association analysis of plant architecture traits using doubled haploid lines derived from different cycles of the Iowa Stiff Stalk Synthetic maize population
Source: Front Plant Sci. 2023 Dec 4;14:1294507. doi: 10.3389/fpls.2023.1294507 (PMC10792766; doi:10.3389/fpls.2023.1294507)
Supplement: Supplementary file 1 [file DataSheet_1.pdf]

Supplementary Material

Genome-wide association analysis of plant architecture traits using doubled haploid lines derived from different cycles of the Iowa Stiff Stalk Synthetic maize population

1 Supplementary Tables

Supplementary Table 1. Combination of random effects with the lowest Bayesian Information Criterion (BIC) values, which were used in the final model to analyze the phenotypic data. Bold values indicate the best fitted model for each trait.

	Random effects						Traits evaluated							
**Model**	**G_j**	**GE_{ij}**	**$ED(G)_{ijk}$**	**$A(ER)_{nil}$**	**$P(ER)_{mil}$**	**Homoscedasticity**	**MAFL**	**FEFL**	**ASI**	**PLHE**	**EAHE**	**FLA**	**TALE**	**NPTB**
1	×	×	×	×	×	No	**6124**	**7007**	4867	19432	19076	18811	11992	10140
1	×	×	×	×	×	Yes	6191	7029	4951	**19419**	**19073**	18804	11982	10140
2	×	×	×	×		No	6357	7160	4861	19620	19111	18803	11985	10132
2	×	×	×	×		Yes	6492	7238	4951	19613	19113	18796	**11975**	**10132**
3	×	×	×		×	No	6337	7147	4866	19627	19111	18806	12017	10165
3	×	×	×		×	Yes	6379	7165	4950	19612	19108	18800	12006	10166
4	×	×		×	×	No	6314	7180	5012	19493	19091	18805	12031	10159
4	×	×		×	×	Yes	6388	7210	5098	19479	19090	18798	12017	10160
5	×	×	×			No	6538	7281	**4860**	19770	19140	18798	12009	10157
5	×	×	×			Yes	6619	7334	4950	19760	19141	18792	11998	10158
6	×	×		×		No	6490	7301	5006	19675	19127	18797	12024	10152
6	×	×		×		Yes	6628	7384	5096	19667	19130	18790	12011	10152
7	×	×			×	No	6488	7299	5015	19671	19124	18800	12056	10183
7	×	×			×	Yes	6539	7324	5100	19655	19124	18794	12043	10185
8	×	×				No	6644	7409	5008	19813	19154	18792	12049	10175
8	×	×				Yes	6735	7470	5098	19800	19158	**18786**	12035	10177
9	×		×			No	6650	7322	4919	19765	19133	18865	12026	10149

Continuation:

9	×	×			Yes	6716	7376	5014	19754	19134	18857	12015	10150
10	×		×		No	6502	7302	5034	19667	19119	18823	12023	10144
10	×		×		Yes	6641	7387	5126	19659	19122	18816	12010	10144
11	×			×	No	6701	7387	5097	19668	19118	18873	12081	10175
11	×			×	Yes	6718	7407	5203	19653	19117	18865	12066	10177
12	×				No	6833	7477	5089	19810	19147	18865	12073	10167
12	×				Yes	6883	7538	5198	19797	19151	18857	12058	10169

MAFL - male flowering, FEFL - female flowering, ASI - anthesis–silking interval, PLHE - plant height, EAHE - ear height, FLA - flag leaf angle, TALE - tassel length, NPTB - number of primary tassel branches.

G_j = the effect of the group of DH line *j;* GE_{ij} = the effect of the interaction between group *j* and environment *i;* $ED(G)_{ijk}$ = the effect of the interaction of environment *i* and DH line *k* within the group of DH line *j;* $A(ER)_{nil}$ = the effect of the range *n* within the environment *i* and replication *l;* $P(ER)_{mil}$ = the effect of the pass *m* within the environment *i* and replication

Supplementary Table 2. Trait BLUPs of flowering and plant architecture traits for 487 BSSS double haploid lines (DHL).

No	Group	DHL	MAFL	FEFL	ASI	PLHE	EAHE	FLA	TALE	NPTB
1	C0	C0_DHL001	65.6	68.4	-2.8	158.9	95.1	37.2	43.8	22.9
2	C0	C0_DHL002	65.4	65.5	-0.1	160.4	76.1	24.4	36.6	13.3
3	C0	C0_DHL004	69.4	71.2	-1.7	180.3	96.9	34.9	30.5	17.5
4	C0	C0_DHL005	66.7	68.4	-1.6	192	103	68	41.6	22.8
5	C0	C0_DHL006	72.1	72.6	-0.6	199.5	81.1	47.7	39	2.5
6	C0	C0_DHL007	64.8	65.1	-0.4	179.6	94.4	62.7	39.1	20
7	C0	C0_DHL008	66.1	67	-0.8	176.5	67	61.2	37.9	17.9
8	C0	C0_DHL010	64.8	67.1	-2.1	158.5	64.7	45.4	42.5	16.6
9	C0	C0_DHL011	67	67.8	-0.9	148.5	76.5	39.4	37.9	9.9
10	C0	C0_DHL013	70	72.1	-2	156.1	71.5	34.9	40.6	17.9
11	C0	C0_DHL014	71.1	70.5	0.3	185.1	94.8	43.2	43.3	14.1
12	C0	C0_DHL015	69.7	69.8	-0.6	190.5	93.9	50.3	39	21.9
13	C0	C0_DHL016	67.7	66.4	0.9	145.4	67.9	43.9	35.2	15.2
14	C0	C0_DHL017	68.4	71.6	-3.2	178.7	80.6	60.5	37.7	6.3
15	C0	C0_DHL018	67.1	71.8	-4.4	175.7	50.4	34.9	39.4	22.8
16	C0	C0_DHL019	65.5	66.9	-1.4	165.7	70.6	28.1	37.6	11.8
17	C0	C0_DHL020	68.4	71	-2.6	188.7	93.6	25.1	33.6	14
18	C0	C0_DHL021	64.7	69.3	-4.2	181.2	96.3	30.4	41.7	13.9
19	C0	C0_DHL022	69.9	71	-1.5	172.8	87.4	31.9	38.2	12.4
20	C0	C0_DHL023	67	64.9	1.5	176.7	90.7	27.4	41.2	16
21	C0	C0_DHL024	67.6	67.8	-0.5	163.4	76.5	28.1	30.7	10.7
22	C0	C0_DHL025	66.7	68.6	-1.8	172.4	83.6	35.7	33.4	10.8
23	C0	C0_DHL026	66.7	67.5	-1	168.6	74.9	21.2	35.7	21.4
24	C0	C0_DHL027	68.1	71.8	-3.4	159.3	84.6	28.9	42.1	23.9
25	C0	C0_DHL028	66.2	67.5	-1.3	157.1	65	42.4	40.1	23.6
26	C0	C0_DHL029	67.9	69.5	-1.7	174.8	84.2	34.9	33.8	21.5
27	C0	C0_DHL032	70.5	71	-0.7	164.8	98.9	42.4	30.7	21.1
28	C0	C0_DHL033	64.6	63.9	0.4	156	70.6	22.9	36.3	21.3
29	C0	C0_DHL034	68.3	70.1	-1.8	199	99.3	31.9	33.9	17.1
30	C0	C0_DHL035	67.4	68.1	-0.9	149.6	70.3	34.2	30.5	13.6
31	C0	C0_DHL036	65.6	64	1.2	168.8	85	56.7	40.4	16.4
32	C0	C0_DHL037	69.5	71.7	-2.2	174.8	93	53	37	16.5
33	C0	C0_DHL038	64.5	65.6	-1.2	142.7	60	36.9	36.9	10.2
34	C0	C0_DHL039	68.6	70.6	-1.9	168.8	82.4	53	39.4	13.7
35	C0	C0_DHL040	70.4	73.1	-2.7	190.9	102.4	46.9	33.7	13
36	C0	C0_DHL041	65.8	65.5	0.2	157.9	76.7	44.7	30	14.5
37	C0	C0_DHL042	67.5	69.7	-2	163.7	76.8	56.7	38.5	19.3
38	C0	C0_DHL043	70.8	72.5	-1.6	161.7	98.9	39.4	36	14.8
39	C0	C0_DHL044	67.3	69	-1.7	163	91.4	59	32.8	22.2
40	C0	C0_DHL045	67.5	70.1	-2.5	182.7	98.8	51.5	39.8	12.4
41	C0	C0_DHL046	70.6	72.5	-1.9	165.4	100	37.2	35.8	14.1

42	C0	C0_DHL047	60.3	63.3	-3	149.7	62.8	33.4	38.4	18.2
43	C0	C0_DHL048	65.8	68.4	-2.3	173.4	76.4	44.7	36.4	18.3
44	C0	C0_DHL049	70.9	72.3	-1.3	178.4	97.4	52.2	42.4	13.9
45	C0	C0_DHL050	72.2	72.2	-0.1	147.4	71	35.7	29.8	7.2
46	C0	C0_DHL051	64	65.5	-1.5	173.4	87.7	89.7	38.5	17.7
47	C0	C0_DHL052	64	66.7	-2.6	161.1	71.4	39.4	38.8	22.2
48	C0	C0_DHL053	67.3	70.3	-2.8	177.3	89.3	42.4	35	8.6
49	C0	C0_DHL054	62.5	64.9	-2.1	185.8	105.5	43.9	40.5	13.1
50	C0	C0_DHL055	66.8	67.3	-0.4	164.7	74.7	35.7	34.7	10.8
51	C0	C0_DHL056	63.9	64.3	-0.4	184.6	84.8	40.9	43.7	12.9
52	C0	C0_DHL058	67.6	69.6	-1.8	159.5	71.2	25.9	36.2	16.3
53	C0	C0_DHL059	70.8	72.2	-1.5	174.6	87.7	26.6	39	11
54	C0	C0_DHL060	66.4	67.3	-0.9	186.5	87.8	64.2	40.3	7.6
55	C0	C0_DHL061	70	73.4	-3.2	181.2	89.6	38.7	36.6	14.5
56	C0	C0_DHL062	72.3	73.6	-1.4	162.7	91	39.4	33.9	15.3
57	C0	C0_DHL063	68.4	70.9	-2.5	184.2	100.3	53	27.9	19.2
58	C0	C0_DHL064	60.8	60.6	-0.2	148.6	67.8	88.8	33.2	14.1
59	C0	C0_DHL065	65.1	68.7	-3.2	180.9	112.5	47.7	37.8	24.7
60	C0	C0_DHL066	68.1	70	-1.9	185	87.6	34.9	36.3	11.8
61	C0	C0_DHL067	71.2	72.1	-0.9	190.1	101.3	55.2	38.1	19.5
62	C0	C0_DHL068	66	67.1	-1.2	177.4	95.9	33.4	44.6	12.3
63	C0	C0_DHL069	64.7	64.4	0	132.1	58.3	50	32.8	13.7
64	C0	C0_DHL071	66.6	68.6	-1.9	191.9	106.4	42.4	34.9	11.5
65	C0	C0_DHL072	67.2	68.6	-1.4	116.4	61.4	37.2	38.8	23.1
66	C0	C0_DHL073	66.7	67.7	-1	163.4	75.2	27.4	44.9	14.8
67	C0	C0_DHL075	68.1	69.3	-1.1	189.9	92.3	42.4	39	21.4
68	C0	C0_DHL076	65.5	67	-1.5	164.8	74.2	33.4	34.3	9.5
69	C0	C0_DHL077	72.1	73.6	-1.4	194.5	108.9	41.7	31	11.1
70	C0	C0_DHL078	68.4	72.3	-3.8	185.6	61.9	33.4	36.2	20.4
71	C0	C0_DHL080	67.8	70.4	-2.5	178.2	94.1	37.9	38.5	17.8
72	C0	C0_DHL081	65.2	68.5	-3	152.9	76.6	31.9	38.5	14.1
73	C0	C0_DHL082	64.6	67.9	-3	180	96.1	59	35.8	16.5
74	C0	C0_DHL083	69.9	73.8	-3.8	182.4	90.8	52.2	38.1	18.5
75	C0	C0_DHL085	66.1	66.2	-0.3	197.8	102.1	37.9	40.8	15.4
76	C0	C0_DHL086	68.2	70	-1.6	150.1	71	42.4	33.6	15.9
77	C0	C0_DHL087	70.3	71.4	-1.1	177.3	103.8	39.4	37.1	18
78	C0	C0_DHL088	71.3	73.9	-2.7	172.5	102.7	34.9	32.1	11.3
79	C0	C0_DHL089	69.4	71.8	-2.4	209.7	136.6	35.7	37.1	14.6
80	C0	C0_DHL090	67.8	70.1	-2.4	150.3	65.2	37.9	39.9	17.7
81	C0	C0_DHL091	68.7	69.9	-1.2	181.3	85.7	51.5	37.7	14.6
82	C0	C0_DHL092	68.6	70.2	-1.5	183.1	103	59.7	37.4	18.9
83	C0	C0_DHL093	70.6	71.4	-0.9	158	75.6	50.7	36.4	9.4
84	C0	C0_DHL094	70	72.7	-2.3	159.8	81.3	37.2	36.5	10.6
85	C0	C0_DHL095	67.1	68.7	-1.6	181.4	90.4	56	43.4	11.9

86	C0	C0_DHL096	66.9	70.8	-3.7	150	69.8	32.7	42.1	19.7
87	C0	C0_DHL098	67	69.6	-2.5	166.2	74.7	15.2	31.7	15.4
88	C0	C0_DHL099	67.8	70.5	-2.7	168.6	93.8	39.4	33.6	15.6
89	C0	C0_DHL100	63.2	63.9	-0.6	134.1	52	37.2	32.5	22.8
90	C0	C0_DHL102	62.6	66.3	-3.4	181.2	70.4	40.2	35.1	29.8
91	C0	C0_DHL103	66.8	68.2	-1.6	151.8	80.7	39.4	27.7	18.9
92	C0	C0_DHL104	65.6	67	-1.5	175.4	82.4	43.9	37.8	6.9
93	C0	C0_DHL106	67.5	67.9	-0.5	153.2	73.6	31.9	32.1	14.9
94	C0	C0_DHL107	63.4	67	-3.3	143.4	48.1	38.7	33.3	17.3
95	C0	C0_DHL108	66.1	67.5	-1.6	149.4	67.9	43.9	29.7	10.1
96	C0	C0_DHL109	67.3	68.6	-1.5	161.2	76	47.7	35.4	12
97	C0	C0_DHL110	69	71	-1.8	148.9	71.2	40.9	37.3	26.1
98	C0	C0_DHL111	69.8	73.2	-3.2	168.6	80.5	52.2	40	14.5
99	C0	C0_DHL112	65.4	69.8	-4	158.7	84.9	41.7	35.8	18.1
100	C0	C0_DHL114	69.4	71.4	-2.1	160.6	70.7	59	39	11.5
101	C0	C0_DHL115	67.9	71.1	-3.3	171	79.6	41.7	46.5	17.9
102	C0	C0_DHL116	64.6	64.7	-0.4	165.4	79.4	23.6	32.1	16.4
103	C0	C0_DHL117	66	67.3	-1.5	179.1	92.2	43.2	43.6	10.9
104	C0	C0_DHL118	70	73	-3	151.7	66	41.7	39.3	23.1
105	C0	C0_DHL119	69.2	72.4	-3	185.9	81.7	15.1	37.2	11.5
106	C0	C0_DHL120	64.4	66.2	-1.7	151.1	74.3	59.7	38.1	14.3
107	C0	C0_DHL121	66.8	71	-3.7	170.1	98.7	46.2	36.4	18.8
108	C0	C0_DHL122	68.6	73.1	-4.3	174.6	72.2	30.4	41.9	14.3
109	C0	C0_DHL123	70	72.9	-2.8	206.2	108.9	44.7	41.4	15
110	C0	C0_DHL124	68	68.4	-0.7	166.7	92.9	37.2	35.9	25.2
111	C0	C0_DHL125	66.8	67.1	-0.5	174.4	77.4	51.5	40.7	8.1
112	C0	C0_DHL127	69.4	70.9	-1.6	159.4	83.6	46.9	37.9	15.7
113	C0	C0_DHL128	63.3	63.6	-0.4	170.5	78.4	41.4	45.1	13.6
114	C0	C0_DHL130	64.6	65.1	-0.7	158.7	69.8	32.7	32.1	7
115	C0	C0_DHL131	66.8	68	-1.4	159	84.7	51.5	28.4	12.2
116	C0	C0_DHL132	70.2	72.8	-2.6	179.1	84.2	46.2	34.7	6.8
117	C0	C0_DHL133	69.8	71.6	-1.7	172.4	91.2	25.9	31.6	13.3
118	C0	C0_DHL134	65.2	65.8	-0.7	180.4	96	38.7	37.6	15.3
119	C0	C0_DHL135	69.7	71.7	-1.9	172.2	97.6	33.4	37	14
120	C0	C0_DHL136	68.8	68.5	0	191.5	102.5	46.2	34.9	8.2
121	C0	C0_DHL137	65.5	67.1	-1.6	196.1	95.7	74	37.9	15.5
122	C0	C0_DHL138	65	66.3	-1.3	181.2	89.9	49.2	32.3	13.7
123	C0	C0_DHL139	66.5	68.9	-2.3	170	84.7	53.7	37.9	15.6
124	C0	C0_DHL140	64.2	65.7	-1.4	156.8	81.1	35.7	33.6	19.4
125	C0	C0_DHL141	62.9	66.2	-3.1	139.5	55.1	22.9	33	18.1
126	C0	C0_DHL143	69.4	72	-2.6	169.3	74.2	46.2	38.8	11.5
127	C0	C0_DHL144	68.6	70.4	-1.8	174.5	94.9	45.4	39.4	13.4
128	C0	C0_DHL145	69.3	71.5	-2.2	133.5	66.2	64.2	34.3	15.9

129	C0	C0_DHL146	66.3	71.2	-4.5	186.4	94.4	45.4	39.4	22.3
130	C0	C0_DHL147	64.8	66.2	-1.5	166.1	72.9	37.9	34.8	17.1
131	C0	C0_DHL148	66.4	68.6	-2	166.9	83.1	40.9	42	12
132	C0	C0_DHL149	74	74.6	-0.9	150.5	71.5	31.2	32.5	12.5
133	C17	C17_DHL001	63.6	64.1	-0.4	183.5	85.6	12.7	43.3	5.5
134	C17	C17_DHL003	67.6	65.5	1.8	198.4	96.3	25.4	37.2	8.6
135	C17	C17_DHL004	63.3	61.8	1.3	134.1	58.8	13.3	37.2	5.1
136	C17	C17_DHL005	61.6	60.4	0.9	171.7	71.8	13.1	40.2	7.6
137	C17	C17_DHL006	61.8	61.7	0.1	190.6	76.7	14.2	43.9	11
138	C17	C17_DHL010	62.9	61.6	1.1	165.4	74.4	6.9	41.4	7.2
139	C17	C17_DHL011	60.5	60.1	0.2	170.9	72.7	23.2	47	5.2
140	C17	C17_DHL013	64.3	65.5	-1	163.4	66.8	35.2	44.6	8.4
141	C17	C17_DHL014	60	59.3	0.5	170.8	65.7	16.3	38.3	7.9
142	C17	C17_DHL018	63.5	62.2	1.2	178.4	82	8.6	42.2	5.7
143	C17	C17_DHL019	64	64.6	-0.6	173.2	58.3	12.4	42	9.7
144	C17	C17_DHL020	61.3	60.7	0.4	186.5	68.4	10.1	46	9.4
145	C17	C17_DHL021	65	64.8	0.2	144	60.5	18.7	40.8	4.6
146	C17	C17_DHL022	61.4	60.7	0.6	172.1	65.1	19.4	39.4	7
147	C17	C17_DHL024	63.7	61.7	1.7	179.2	83.4	18.7	42.7	10.1
148	C17	C17_DHL026	58.9	58.8	0.1	162.4	63.1	7.1	40.1	4.2
149	C17	C17_DHL029	60.2	60.2	-0.1	153	48.4	25.4	36	6.5
150	C17	C17_DHL031	61.9	61.2	0.5	171.1	55.1	12.4	40.4	5.6
151	C17	C17_DHL034	62.8	62.2	0.5	165.9	58.9	7.1	51.1	7.5
152	C17	C17_DHL035	61.8	61.4	0.5	177.5	75.2	14.8	41.9	5
153	C17	C17_DHL036	65.4	64.9	0.4	188.7	94.4	10.3	39.5	10
154	C17	C17_DHL038	62.7	62.9	-0.1	162.6	55.8	11.2	41.1	8.9
155	C17	C17_DHL041	61.8	63.2	-1.1	171.7	69.8	5.1	42.2	9.7
156	C17	C17_DHL043	61.6	63	-1.2	148	59.3	15.7	39	6.5
157	C17	C17_DHL044	63.2	63	0.1	177.4	67.1	8.2	44.4	8.7
158	C17	C17_DHL045	59.5	60.1	-0.4	169.1	58.9	6.5	46.8	7.1
159	C17	C17_DHL046	61.6	61.7	0	170	63.8	14.8	47.2	5.4
160	C17	C17_DHL050	63	65.2	-1.9	181.8	79.1	23.9	38	9.3
161	C17	C17_DHL052	64	63.7	0.4	203.2	83.5	18.5	47.2	11.4
162	C17	C17_DHL053	64.3	63	1.1	172.3	78.3	24.7	39.3	8
163	C17	C17_DHL054	60.6	60.4	0.3	158.3	58.6	5.3	53.3	2.9
164	C17	C17_DHL055	67.4	65.2	1.8	164.5	71.7	19.4	40	5.9
165	C17	C17_DHL056	61.9	61	0.6	154.7	62.4	19.4	44.2	6
166	C17	C17_DHL058	64.4	64.4	0.1	182.3	73.4	6.2	47.8	5.9
167	C17	C17_DHL061	61.8	61.7	0.1	150.6	52.5	9.5	41.3	5.5
168	C17	C17_DHL062	64.4	64.8	-0.3	179.8	62.9	14.2	37.5	11.2
169	C17	C17_DHL064	64.3	66.2	-1.5	165	53.6	9.5	36.6	5.9
170	C17	C17_DHL066	65.8	66.3	-0.4	175.5	78.2	10.9	42.9	9
171	C17	C17_DHL067	61.2	61	0.2	164	57.7	38.2	36.1	11.3
172	C17	C17_DHL071	65.7	64.8	0.8	148.8	66.5	5	46.4	4.2

173	C17	C17_DHL072	60.6	60.6	0.1	184.3	74.9	6.5	40.8	7.9
174	C17	C17_DHL074	65	66.5	-1.2	182.9	71.6	10.9	42.1	5
175	C17	C17_DHL077	62.6	61.4	1.2	151.8	58.9	20.9	45.8	8.3
176	C17	C17_DHL078	60.3	60.7	-0.5	167.8	67.5	10.3	46.1	8.5
177	C17	C17_DHL079	62.4	62	0.3	157.7	71.4	13.4	43	4.2
178	C17	C17_DHL080	63.2	62.5	0.6	170.7	63.6	11.2	44	7.5
179	C17	C17_DHL082	63.1	64.9	-1.5	180.7	75.4	10.4	39.7	9
180	C17	C17_DHL087	63.9	63.8	0.1	163.1	67.2	8.6	41.8	6.2
181	C17	C17_DHL089	59.4	59.4	-0.1	169.3	74.6	9.2	51.3	6.1
182	C17	C17_DHL090	62.8	62.4	0.3	171.1	72.3	29.2	47.4	6.6
183	C17	C17_DHL091	64.6	64.3	0.4	174.3	70.6	17.2	43.1	4.4
184	C17	C17_DHL093	61	60.6	0.3	165.1	59.6	14.2	37.3	5
185	C17	C17_DHL098	63.3	63.1	0	175.9	72.5	9.5	36.2	7
186	C17	C17_DHL102	61	60.1	0.7	173.3	72.7	23.2	34.8	8.3
187	C17	C17_DHL103	61.3	63.3	-1.6	157.2	52.5	5.4	44	5.6
188	C17	C17_DHL105	63	62.7	0.3	174.5	71.6	16.4	39.8	7.4
189	C17	C17_DHL106	59.2	60.6	-1.3	167.6	56.2	9.7	42.8	10.8
190	C17	C17_DHL107	64.7	65.2	-0.9	189.1	76	14.2	43.3	5.5
191	C17	C17_DHL108	67.4	65.7	1.6	181.1	92.7	7.7	41.4	13.4
192	C17	C17_DHL109	64.4	62.6	1.6	169	65.5	13.1	40.3	3.8
193	C17	C17_DHL110	64.3	64.4	0	181.2	72.7	27.7	37.9	14.2
194	C17	C17_DHL111	61.5	62.4	-1	163.6	63.3	6.9	48.3	5.1
195	C17	C17_DHL113	60.6	60.6	0	174.3	73.5	23.2	42.8	7.6
196	C17	C17_DHL115	65.8	62.9	2.4	182.3	95.6	8.6	46.5	7.5
197	C17	C17_DHL116	62.6	63.6	-0.9	170.3	66.9	18.4	43	12.6
198	C17	C17_DHL118	64	63.3	0.5	179.5	77.9	10.3	46	7.4
199	C17	C17_DHL119	64.1	63.9	0.1	171.7	67.5	6	43.3	8.6
200	C17	C17_DHL120	63.9	64	0.1	151.5	59.5	17.2	41.6	10.7
201	C17	C17_DHL121	62.7	61.3	1.3	157	64.2	13.1	43	6.3
202	C17	C17_DHL124	59.8	59.7	-0.1	170.9	67.5	10.1	42	8
203	C17	C17_DHL126	65.4	65	0.4	201.2	86.9	15.7	39.3	6.9
204	C17	C17_DHL127	62.8	63.7	-0.8	167.8	68.5	10.4	46	8
205	C17	C17_DHL128	63	64.2	-0.9	163.9	62.2	14.9	46.1	6.7
206	C17	C17_DHL129	64.5	65.1	-0.4	158.9	70.8	12.7	39.2	7.5
207	C17	C17_DHL132	63.1	62.2	0.7	166.9	68.8	11.9	44.8	4.6
208	C17	C17_DHL134	62	62.9	-0.8	161.4	58.9	6.9	40.8	5.5
209	C17	C17_DHL135	60.8	61.7	-0.7	126.6	44.2	12.5	39.5	7.5
210	C17	C17_DHL136	64.7	64.9	-0.2	196.9	72.2	14.8	46.8	5
211	C17	C17_DHL138	61.3	60.2	0.8	183.1	67.3	8.2	41.9	5.7
212	C17	C17_DHL139	64.9	64.4	0.4	165.5	71.9	12.7	47.2	4.3
213	C17	C17_DHL140	66.4	64.9	1.4	186	69.6	11	42.6	7.9
214	C17	C17_DHL142	61.5	60.6	0.9	149.9	53.8	14.2	38.3	8.3
215	C17	C17_DHL143	60.5	61	-0.6	138.2	40.6	11.2	45.3	7.9

216	C17	C17_DHL144	65.4	65.6	-0.1	162.8	58.8	6.2	40.8	7.8
217	C17	C17_DHL146	62.6	63.4	-0.8	174.9	71.2	11	38.9	7.9
218	C17	C17_DHL147	62.4	62.7	-0.2	169.9	65	14.9	45	9.3
219	C17	C17_DHL150	62.2	62.6	-0.2	176.7	67.8	24.7	37	9.6
220	C17	C17_DHL152	64.5	63.2	1.1	164.1	69.6	8.6	45.4	7.2
221	C17	C17_DHL153	65.6	66.6	-1	175.2	74.2	11.9	43.2	6.6
222	C17	C17_DHL154	61.8	61.3	0.3	168.4	69.5	17.8	39.5	8.3
223	C17	C17_DHL155	65.5	64.9	0.5	193.3	92.5	21.7	40.1	10.5
224	C17	C17_DHL158	63.7	64.7	-0.9	178	76.9	12	43.5	8.5
225	C17	C17_DHL160	61.7	61.1	0.6	142.2	49.2	12.7	38.9	7.6
226	C17	C17_DHL161	63.8	63.1	0.6	156.4	60.7	17.9	42.8	6.2
227	C17	C17_DHL162	61.7	63.2	-1.2	182.9	67.5	5.3	38.2	4.4
228	C17	C17_DHL163	63.9	64.1	-0.2	167.2	78.3	10.3	43.3	8.1
229	C17	C17_DHL165	60.1	60.3	-0.1	158.9	62.4	25.4	48.4	7.4
230	C17	C17_DHL166	64.3	66.3	-1.5	188.7	82.8	5.4	42.1	8.1
231	C17	C17_DHL167	65.9	65.7	0.2	193.6	89.5	17.2	39.7	5.2
232	C17	C17_DHL169	63.1	63.9	-0.6	182.9	81.8	9.5	42.5	4.6
233	C17	C17_DHL170	58.6	58.9	-0.1	141.7	51.3	12.5	41.9	5.5
234	C17	C17_DHL172	66.7	65.3	1.2	176.5	72.6	8	42.7	4.9
235	C17	C17_DHL174	61.2	61.2	-0.1	175.8	64.4	18.7	41	5.6
236	C17	C17_DHL175	60.2	59.6	0.7	153.2	58.2	35.2	35.9	5.8
237	C17	C17_DHL177	62.7	64	-0.5	171.6	66.3	11.6	43.6	5.1
238	C17	C17_DHL179	62.2	62.7	-0.2	179.7	72.4	15.7	45.9	8.8
239	C17	C17_DHL180	62.1	62.8	-0.6	180.5	67.9	7.7	39.2	9
240	C17	C17_DHL181	66.4	65.4	1	182.9	75.7	8.6	39.7	5.8
241	C17	C17_DHL184	63.5	63.4	0.1	155.2	74.2	10.1	45	5
242	C17	C17_DHL188	63.5	62.3	1	183.5	70.4	13.4	44.4	5.5
243	C17	C17_DHL189	60.2	58.9	1.2	184.9	84.5	12.5	45.9	4.5
244	C17	C17_DHL190	60.5	60.1	0.4	151.2	54.7	6.2	47.7	5.2
245	C17	C17_DHL191	60.1	58.9	1	166.4	68.2	23.9	38.2	11.7
246	C17	C17_DHL195	60.8	60.6	0.1	172.9	61	6.8	39.5	5.4
247	C17	C17_DHL196	64	65.7	-1.6	160.6	49.9	14.8	34.9	5.1
248	C17	C17_DHL202	64.3	64.2	0.2	166.7	66.8	16.4	38.2	3.9
249	C17	C17_DHL205	61.8	62.2	-0.1	152.6	66.3	23.9	41.9	9
250	C17	C17_DHL210	61.4	61.5	-0.1	181.4	81.3	11.6	46.8	5.9
251	C17	C17_DHL216	61	62.2	-1.1	166.1	68.4	18.7	35.7	8.6
252	C17	C17_DHL217	64.2	64	0.2	169.6	63.5	7.1	50.5	5.5
253	C17	C17_DHL218	61.2	60.9	0.2	177.9	77.9	10.1	40	6.2
254	C17	C17_DHL219	59.2	58.6	0.4	165.5	53.5	12.5	44.8	9.7
255	C17	C17_DHL220	60.7	60.8	-0.1	166.1	73.4	21.7	46.4	5.5
256	C17	C17_DHL221	61	59.9	0.9	178	67.8	19.3	39.3	6.5
257	C17	C17_DHL223	60.2	59.9	0.4	154.1	60	11.2	36.7	6.7
258	C17	C17_DHL224	63.3	62.9	0.4	157.5	58.7	11.2	38.7	6.4
259	C17	C17_DHL225	59.9	60.3	-0.4	156.5	62.2	10.1	39.6	4.5

260	C17	C17_DHL226	64.9	64.3	0.6	196	81.8	20.2	39	6
261	C17	C17_DHL228	60.9	61.3	-0.2	173.3	53.8	7.7	41.9	5.3
262	C17	C17_DHL232	64.2	63.6	0.6	187	71.1	5.1	45.2	5.8
263	C17	C17_DHL233	63	63.6	-0.6	166	66.5	11.2	46.8	4.1
264	C17	C17_DHL236	61.8	62.7	-0.7	207.3	96	14.2	42.4	10.9
265	C17	C17_DHL238	61.4	60.8	0.4	171.1	64.7	8.9	39	6.2
266	C17	C17_DHL239	60.9	59.1	1.7	169.9	74.4	10.1	36.8	5.1
267	C17	C17_DHL240	61.8	63.5	-1.3	167.9	62.1	5.3	46.8	7.4
268	C17	C17_DHL241	62.3	62.6	-0.2	155	62.8	10.3	43.6	4.5
269	C17	C17_DHL243	59.1	58	0.8	171.1	69.1	20.2	41.7	6.6
270	C17	C17_DHL244	61.9	62.9	-0.8	178.5	64.9	11.6	44.3	4
271	C17	C17_DHL247	63.9	64.7	-0.6	186	74.7	8	42.5	6
272	C17	C17_DHL248	60.6	59.5	1.1	147.2	46.2	6.5	39.1	6.5
273	C17	C17_DHL252	63.7	64.7	-0.8	192.7	57.6	11.5	46.9	3.6
274	C17	C17_DHL253	63.9	65.2	-1	142.1	56.4	11.2	40.2	5.1
275	C17	C17_DHL255	63.6	62.7	0.7	171.2	77	19.4	38.3	12
276	C17	C17_DHL258	63.1	64.6	-1.3	174.8	71	27.7	44.5	9.6
277	C17	C17_DHL259	62.8	63.6	-0.7	168.6	62	11	51.2	8.7
278	C17	C17_DHL262	61.5	59.8	1.4	172.6	71	14.2	37.7	7
279	C17	C17_DHL264	61.5	61.1	0.4	166.4	69.5	24.7	37.1	9.5
280	C17	C17_DHL265	64.5	63.7	0.7	168.7	80.6	6.5	44.3	9.5
281	C17	C17_DHL267	62.3	63	-0.5	181	73	14.8	40.9	6
282	C17	C17_DHL268	63.2	61.7	1.4	170.3	77	16.4	44.3	6.8
283	C17	C17_DHL270	64.3	65	-0.7	179.2	84.2	23.9	44.4	11.5
284	C17	C17_DHL271	66	66.3	-0.2	171.9	80.2	27	39.5	7.2
285	C17	C17_DHL273	59.8	59.9	0.1	166.3	66.3	16.3	39.9	7.4
286	C17	C17_DHL280	64.8	64.5	0.3	171.9	61.6	25.4	41	7
287	C17	C17_DHL282	59.4	59.7	-0.2	169	63.6	17.2	42	6.4
288	C17	C17_DHL283	64.4	64.8	-0.3	172.1	83.7	19.4	41.4	15.9
289	C17	C17_DHL284	68.4	70.4	-1.8	192.1	93.6	27	41.5	7.5
290	C17	C17_DHL286	69	67.5	1.4	199.5	90.7	13.3	41.6	5.3
291	C17	C17_DHL287	62	62.3	-0.4	159	52.8	6.3	42.3	8.2
292	C17	C17_DHL290	61.6	62.5	-0.8	168.1	65.3	10.9	46.5	5.8
293	C17	C17_DHL294	63.3	63.2	0	152.3	53.2	6.9	41.7	6.8
294	C17	C17_DHL296	61.3	60.3	1	170.8	65.6	18.7	42.6	9.2
295	C17	C17_DHL297	61.7	60.3	1.3	175.9	70.6	19.4	36.8	3.8
296	C17	C17_DHL298	60.9	60.6	0.3	162.9	65	12.7	44.8	7.7
297	C17	C17_DHL299	65.1	65.3	0	155.5	61.1	6.3	39.8	4.9
298	C17	C17_DHL301	63.6	62.6	0.8	178.2	82.3	16.4	42.5	10.5
299	C17	C17_DHL304	60.7	59.8	0.6	169.5	56.4	26.2	35.5	6.1
300	C17	C17_DHL305	62.7	63.1	-0.3	162.4	55.6	8.8	38	6.2
301	C17	C17_DHL307	66.2	65.9	0.3	197.2	98.7	8	49.3	12
302	C17	C17_DHL308	61.6	62.2	-0.6	166.8	57.6	20.2	41.6	7.9

303	C17	C17_DHL309	63.2	65	-1.6	179.2	68.3	11.2	46	6
304	C17	C17_DHL310	62.7	63	-0.4	163.9	54.7	17.2	38.4	12.3
305	C17	C17_DHL311	63.6	63.5	0.1	165.8	71.4	7.1	50.1	5
306	C17	C17_DHL312	65.2	65.4	-0.1	184	81.1	20.9	39.2	9.6
307	C17	C17_DHL313	62.1	62.1	-0.1	162.3	68.9	11.2	44.3	9.4
308	C17	C17_DHL315	63.5	63.1	0.3	169	71.8	11.9	40.7	7.6
309	C17	C17_DHL316	60.7	61.9	-0.9	183	77.7	11.2	44.4	9.3
310	C17	C17_DHL317	63.7	64.1	-0.3	161.1	73.8	11	45.8	6.6
311	C17	C17_DHL319	65.1	66.5	-1.1	161.9	57.6	14.9	47	6.1
312	C17	C17_DHL321	60.3	59.6	0.6	163.6	64.5	17.2	40.6	6.9
313	C17	C17_DHL323	63.2	62.7	0.5	161.2	46.8	18.7	43.2	6.9
314	C17	C17_DHL324	63.1	62.6	0.4	154.7	65.9	15.5	36.5	5
315	C17	C17_DHL326	61.1	61.3	-0.1	167.8	70.8	10.1	45.5	8.1
316	C17	C17_DHL327	62.9	61.5	1	168.6	64.1	17.9	40.2	8.6
317	C17	C17_DHL332	61.8	62.1	-0.1	189.9	76.9	8.2	39.3	11.1
318	C0/C17	C0/C17_DHLL001	63.4	63.9	-0.4	141.1	56.6	28.5	36.3	6.3
319	C0/C17	C0/C17_DHLL002	64.7	65.4	-0.7	184.2	85.2	46.6	43.9	10.4
320	C0/C17	C0/C17_DHLL003	64.9	64.4	0.4	176.5	86.3	29.3	40.2	11.9
321	C0/C17	C0/C17_DHLL005	64.5	64.8	-0.3	175.7	78.8	18	39	11.5
322	C0/C17	C0/C17_DHL007	68.2	69.1	-1	165.3	86.9	41.3	37.6	17.4
323	C0/C17	C0/C17_DHL008	66.4	68.1	-1.7	169.6	63.5	24.6	41.9	12.9
324	C0/C17	C0/C17_DHL010	66.3	65.3	0.8	156.9	85.4	47.3	32.5	12.9
325	C0/C17	C0/C17_DHL012	65.6	65.3	0.4	171	80.1	20.3	41.3	7.3
326	C0/C17	C0/C17_DHL013	65	68.4	-3.1	151.5	62.3	53.3	39.2	8.1
327	C0/C17	C0/C17_DHL014	60.9	62.4	-1.4	164.3	60.9	45.8	40.6	10
328	C0/C17	C0/C17_DHL015	65.6	66.5	-0.7	184.8	67.1	18	41.8	9.1
329	C0/C17	C0/C17_DHL016	63.5	64.4	-0.9	175.8	108.7	35.3	41.6	7.8
330	C0/C17	C0/C17_DHL017	65.2	66.5	-1.3	182.3	83.9	12	45.7	6.6
331	C0/C17	C0/C17_DHL018	63.3	64.9	-1.5	129.5	56.4	24.6	33.6	15
332	C0/C17	C0/C17_DHL019	60.6	60.8	-0.2	147.2	59.5	22.8	37.5	12.5
333	C0/C17	C0/C17_DHL020	68.6	71.5	-2.6	172.1	84.1	35.3	35.4	12.7
334	C0/C17	C0/C17_DHL021	67.4	66.5	0.8	157.4	76.4	33.8	33.5	5.8
335	C0/C17	C0/C17_DHL024	64.5	62.5	1.5	174.1	77.3	21	31	9
336	C0/C17	C0/C17_DHL025	64.1	62.2	1.6	181.1	82.5	25.5	35.8	4.8
337	C0/C17	C0/C17_DHL027	63.1	63.7	-0.6	166.7	64	11.2	41.8	5.9
338	C0/C17	C0/C17_DHL030	66.4	65.2	1.1	190.1	85	56.4	45.4	7.3
339	C0/C17	C0/C17_DHL033	63.6	65.7	-1.7	156.6	53.1	16.5	34.9	6.9
340	C0/C17	C0/C17_DHL035	62.8	64.1	-1.3	159.9	68.2	30.8	31	10.8
341	C0/C17	C0/C17_DHL037	68.4	69.5	-1.2	156.5	78.5	27.8	33	14.2
342	C0/C17	C0/C17_DHL038	63.5	64.5	-1	180	76.6	17.8	46.8	5.4
343	C0/C17	C0/C17_DHL040	68.4	70.2	-1.5	198.2	111.8	15.7	44.3	8.4
344	C0/C17	C0/C17_DHL041	61.4	61.9	-0.6	167.4	75.4	18	38.8	8.5
345	C0/C17	C0/C17_DHL042	66.5	67.1	-0.5	161.5	73.3	36.8	36.7	11
346	C0/C17	C0/C17_DHL043	71.1	71.7	-0.8	185.6	94.7	37.6	41.5	18.9

347	C0/C17	C0/C17_DHL044	62.4	63.1	-0.7	171.9	55.8	20.1	44.1	10
348	C0/C17	C0/C17_DHL045	67.1	68.4	-1.2	148.4	66.4	24.8	32.5	11.9
349	C0/C17	C0/C17_DHL047	65.6	66.2	-0.8	189.9	108.9	26.3	47.3	10
350	C0/C17	C0/C17_DHL048	66.3	66.8	-0.6	170.2	89.2	38.3	34.7	13
351	C0/C17	C0/C17_DHL049	63.5	64.6	-1	149.5	72.7	31.5	34.5	10.7
352	C0/C17	C0/C17_DHL051	65.5	69.2	-3.3	158.3	74.8	36.8	39.4	8.2
353	C0/C17	C0/C17_DHL052	62.9	62.6	0.1	188	93.3	54.4	36	11.1
354	C0/C17	C0/C17_DHL053	69.8	71.6	-1.6	187.2	87.7	35.3	42	19.1
355	C0/C17	C0/C17_DHL056	61.7	63.1	-1.3	168.8	80.4	32.3	30.7	10.4
356	C0/C17	C0/C17_DHL058	70.7	68.1	1.2	162.8	76.2	16.5	39.1	10.1
357	C0/C17	C0/C17_DHL059	66.6	69.9	-3	191.7	90.3	34.5	39.3	9.4
358	C0/C17	C0/C17_DHL060	64.7	64.4	-0.1	209.3	95.2	47.3	42.4	12.5
359	C0/C17	C0/C17_DHL061	70.1	70.5	-0.6	188.8	86.2	25.5	28.4	12.6
360	C0/C17	C0/C17_DHL062	62.4	63.6	-1.3	187.7	95.9	32.3	43.8	7.3
361	C0/C17	C0/C17_DHL064	65	66.5	-1.5	149.4	62	30.8	38.6	20.5
362	C0/C17	C0/C17_DHL066	62	60.6	1	168.6	80.9	39.8	34.8	10.1
363	C0/C17	C0/C17_DHL068	66.7	69.6	-2.6	175.2	89.4	20.3	32.1	9.6
364	C0/C17	C0/C17_DHL069	66.1	66.8	-0.5	169.1	77.6	22.5	38.3	10.9
365	C0/C17	C0/C17_DHL070	68.3	68.8	-0.4	172.7	103.5	33.8	34.5	20.4
366	C0/C17	C0/C17_DHL071	66.5	68.1	-1.4	178.8	87.6	27	44.8	12.7
367	C0/C17	C0/C17_DHL072	64.3	65.7	-1.4	179.8	88.4	43.6	36.7	15
368	C0/C17	C0/C17_DHL073	62.3	62.3	0.1	189.1	65	25.5	36.7	8.1
369	C0/C17	C0/C17_DHL074	67.1	67.1	0	176	74.3	27.8	38.5	14.9
370	C0/C17	C0/C17_DHL076	61.2	62.7	-1.4	180.8	71.8	24	44.4	5.9
371	C0/C17	C0/C17_DHL078	64	63.9	-0.1	175.5	67.5	23.3	35.6	11.2
372	C0/C17	C0/C17_DHL079	64.3	64.8	-0.6	190.3	94	47.3	39.8	12.8
373	C0/C17	C0/C17_DHL080	60.6	60.9	-0.4	183.7	67.9	43.6	42.4	5.3
374	C0/C17	C0/C17_DHL081	63.1	65.5	-2.2	161.7	78.1	20.1	43.4	10.5
375	C0/C17	C0/C17_DHL082	66.1	65.2	0.9	171.9	65.4	21.6	36.5	5.9
376	C0/C17	C0/C17_DHL083	70.7	70.7	-0.2	169.3	77.6	16.3	44.5	6.4
377	C0/C17	C0/C17_DHL084	66.3	69.1	-2.8	152.7	47.9	36	37	9.9
378	C0/C17	C0/C17_DHL085	66.1	68.1	-1.9	163.8	82.5	34.5	46.9	9.4
379	C0/C17	C0/C17_DHL086	64.2	64	0.1	179.2	78.9	24	38.2	13.3
380	C0/C17	C0/C17_DHL087	64.7	64.7	-0.2	166.3	81	38.3	42.3	6.3
381	C0/C17	C0/C17_DHL088	66.5	69.4	-3	163.8	65.7	12.7	42.2	6.1
382	C0/C17	C0/C17_DHL089	64.9	66.4	-1.5	185.8	91.2	15	36.2	11.4
383	C0/C17	C0/C17_DHL090	67.8	69.9	-2	164.3	97.9	35.3	27.1	12
384	C0/C17	C0/C17_DHL091	67	68	-1	164.9	77	8.5	40.3	6.5
385	C0/C17	C0/C17_DHL092	61.8	64	-2.2	184.5	73.7	17.2	40.1	10.5
386	C0/C17	C0/C17_DHL094	63.6	64.3	-0.8	144.7	50.3	23.3	35.4	16
387	C0/C17	C0/C17_DHL095	65.1	69.1	-3.8	160.5	73.2	36	44.4	11.9
388	C0/C17	C0/C17_DHL096	65.2	66.6	-1.2	158.4	78.6	37.6	36.9	14.6
389	C0/C17	C0/C17_DHL097	67.7	69.9	-2	184.9	96.2	33.8	41.9	12.1

390	C0/C17	C0/C17_DHL100	67.6	66.7	0.7	184	91	27.8	37.4	8.2
391	C0/C17	C0/C17_DHL103	66.3	66.8	-0.6	168.8	75.3	23.9	36.6	9.5
392	C0/C17	C0/C17_DHL104	67	68.6	-1.5	193.4	88.2	30.8	42.9	9.6
393	C0/C17	C0/C17_DHL105	63	64.9	-1.7	163.3	84.3	39.8	35.9	4.9
394	C0/C17	C0/C17_DHL106	65.4	65.1	0.1	169.1	73.8	20.3	39.7	7.6
395	C0/C17	C0/C17_DHL107	65.2	65.7	-0.4	152.7	62.1	23.3	36.9	12.7
396	C0/C17	C0/C17_DHL108	66.7	67.2	-0.6	171.7	85.6	21	46.2	16.3
397	C0/C17	C0/C17_DHL109	65.6	65.9	-0.2	173.4	82	27.8	35.8	6.6
398	C0/C17	C0/C17_DHL112	67.2	68.3	-1.2	174.7	103.3	42.1	40.1	9.3
399	C0/C17	C0/C17_DHL114	63.9	65	-1	179.6	90.7	51.8	32.4	12.1
400	C0/C17	C0/C17_DHL117	67.7	69.1	-1.4	184	76.5	32.3	43	11.7
401	C0/C17	C0/C17_DHL118	63.7	63.2	0.3	198.1	106.8	23.3	40.9	12.2
402	C0/C17	C0/C17_DHL121	68.1	68.6	-0.6	178.9	78.3	33.8	37.8	9.5
403	C0/C17	C0/C17_DHL122	68.5	70	-1.6	175.3	97.3	72.9	38.6	17
404	C0/C17	C0/C17_DHL125	66.3	67.9	-1.6	198	102.6	30.8	42.2	5.5
405	C0/C17	C0/C17_DHL126	66.4	66.1	0.2	159.7	63.6	20.3	36.3	7.4
406	C0/C17	C0/C17_DHL129	65.8	66.2	-0.5	199.1	98	57.1	39.9	7.6
407	C0/C17	C0/C17_DHL130	66.5	66.9	-0.4	160.3	68.1	24.8	41.8	13.2
408	C0/C17	C0/C17_DHL132	67.5	68.4	-1	162.4	70	58.6	32	19.7
409	C0/C17	C0/C17_DHL136	66.2	67.8	-1.5	163.9	75.2	44.6	31.7	11.3
410	C0/C17	C0/C17_DHL137	64	65.9	-1.7	173	72.8	18	39.5	8.6
411	C0/C17	C0/C17_DHL139	62.1	63.1	-1	176	70.8	24	38.7	10.8
412	C0/C17	C0/C17_DHL140	62.6	63.9	-1.3	154.5	61.5	14.8	39.9	11.3
413	C0/C17	C0/C17_DHL141	63.9	64	-0.1	170.2	79.6	31.5	42.9	13.1
414	C0/C17	C0/C17_DHL142	64.1	66.2	-1.9	176	66.2	31.5	43.5	7.9
415	C0/C17	C0/C17_DHL146	65.8	66.4	-0.6	180.4	81.9	15.6	40.8	7.9
416	C0/C17	C0/C17_DHL147	65.6	65.7	-0.2	221.2	122.2	39.1	46.3	12.4
417	C0/C17	C0/C17_DHL148	67.8	69.4	-1.6	161.4	76.6	17.2	42.8	12.4
418	C0/C17	C0/C17_DHL149	65.9	67.5	-1.6	180.2	95.1	33.8	37.8	10.1
419	C0/C17	C0/C17_DHL150	64.9	64.3	0.3	162.4	70.8	28.5	40.5	11.6
420	C0/C17	C0/C17_DHL152	68	72	-3.7	176.1	81.1	24	46.2	13.8
421	C0/C17	C0/C17_DHL153	63.1	65.7	-2.4	159.3	74.9	35.3	34	7.7
422	C0/C17	C0/C17_DHL154	64.6	67.7	-3	162.3	69.2	30	52.2	8
423	C0/C17	C0/C17_DHL155	65.2	69.8	-4	142.6	44.3	50.3	36.7	6.9
424	C0/C17	C0/C17_DHL156	64.5	65.1	-0.8	160.6	75.9	46.6	39.1	9.4
425	C0/C17	C0/C17_DHL157	65	66.1	-1.2	155	74.5	35.3	34.1	15.8
426	C0/C17	C0/C17_DHL158	63.2	64.6	-1.2	188.7	78.7	33.8	48.2	6.7
427	C0/C17	C0/C17_DHL159	66.2	65.1	0.9	189.8	71.3	28.5	40.6	6.1
428	C0/C17	C0/C17_DHL160	63.7	63.8	-0.1	168.1	74.5	42.1	36.9	6.2
429	C0/C17	C0/C17_DHL161	67.4	68.2	-1.1	202.3	112.3	33	35.8	8.1
430	C0/C17	C0/C17_DHL162	65.2	64.4	0.7	184.8	76.9	26.3	39.5	8.3
431	C0/C17	C0/C17_DHL163	70.1	69.9	0.1	142.1	61.1	40.6	36.4	6.7
432	C0/C17	C0/C17_DHL164	60.8	60.7	0.1	163.7	67.3	19.5	42.3	6
433	C0/C17	C0/C17_DHL165	62.7	62.2	0.3	167.7	88.7	20.3	40.1	13.8

434	C0/C17	C0/C17_DHL166	65.1	66.4	-1.1	149	59.5	17.2	44.1	9.6
435	C0/C17	C0/C17_DHL168	67.7	70.3	-2.4	197	108.2	27.8	37.3	6.8
436	C0/C17	C0/C17_DHL169	65.9	66	-0.1	175	90.6	8.4	38.9	6.6
437	C0/C17	C0/C17_DHL172	62	62.3	-0.3	175.6	77.9	20.9	32.7	8.4
438	C0/C17	C0/C17_DHL174	65.6	65	0.4	166.6	74.6	23.3	42.9	9
439	C0/C17	C0/C17_DHL177	67.2	67.9	-0.7	198.3	105.1	33.5	40.7	22.4
440	C0/C17	C0/C17_DHL178	64.6	63.7	0.8	184.9	85.9	34.5	35.7	7.6
441	C0/C17	C0/C17_DHL183	66.4	65.2	1	178	87.8	35.3	28.6	12.9
442	C0/C17	C0/C17_DHL184	68.9	69.7	-1	175.5	90.7	30.5	34.3	18.1
443	C0/C17	C0/C17_DHL185	67.3	68.3	-1.3	166.2	76.3	25.5	38.7	6.5
444	C0/C17	C0/C17_DHL187	67.5	72.1	-4.4	156.8	60.6	27.8	40.3	14.3
445	C0/C17	C0/C17_DHL188	69.3	70.5	-1.1	164.8	88.3	71.4	41.6	8.1
446	C0/C17	C0/C17_DHL189	66.4	67	-0.8	181.9	76.3	10.2	36.8	4.5
447	C0/C17	C0/C17_DHL190	66.5	69	-2.3	154	77.8	32.3	31.6	6.8
448	C0/C17	C0/C17_DHL191	66.3	67.5	-1.1	146.3	54.3	21.8	41.7	18.5
449	C0/C17	C0/C17_DHL192	64.3	64.9	-0.5	170.8	82	25.5	38.9	10.4
450	C0/C17	C0/C17_DHL193	63.9	64.9	-0.9	164.4	72.8	20.3	45.8	7.9
451	C0/C17	C0/C17_DHL195	68.5	68.7	-0.5	199.4	115.5	33.8	40.2	10.5
452	C0/C17	C0/C17_DHL196	65.9	66.1	-0.4	187.4	99.3	36.8	44.3	11.5
453	C0/C17	C0/C17_DHL197	62.9	62.6	0.2	191.8	87	45.7	44	11.8
454	C0/C17	C0/C17_DHL198	66.3	67	-0.7	170.8	70.2	45.8	38.8	9.5
455	C0/C17	C0/C17_DHL199	62.3	62.1	0	189.3	89.2	54.1	39.2	13.3
456	C0/C17	C0/C17_DHL200	63.9	66	-2	154.4	54.6	30	40.4	15.1
457	C0/C17	C0/C17_DHL201	61.5	62.1	-0.6	202	94.2	48.1	44.1	12.3
458	C0/C17	C0/C17_DHL202	65.6	65.1	0.4	183.7	87.1	30	43.2	9.2
459	C0/C17	C0/C17_DHL203	65.9	66.7	-1	199.1	98.4	46.6	38.9	12.3
460	C0/C17	C0/C17_DHL204	65.2	64.3	0.6	184.1	85.9	39.8	42.7	8.3
461	C0/C17	C0/C17_DHL205	68.3	69.2	-0.8	165	92.9	40.6	36.6	6.5
462	C0/C17	C0/C17_DHL206	67.2	68.3	-1.1	171.3	99.7	29.3	35.2	22
463	C0/C17	C0/C17_DHL207	64.9	65.8	-0.7	147.1	60.8	18.8	40.2	11.5
464	C0/C17	C0/C17_DHL208	65.5	69.8	-3.9	169.8	69.8	34.5	38.7	15.8
465	C0/C17	C0/C17_DHL209	61.8	61.6	0	182.4	71.6	18	46.4	4.9
466	C0/C17	C0/C17_DHL212	64.3	65.9	-1.3	148.4	55	34.5	37.3	10.6
467	C0/C17	C0/C17_DHL213	67.8	69.5	-1.6	151.6	65.5	10.5	35.2	2.9
468	C0/C17	C0/C17_DHL214	68	68.7	-0.7	154.3	68.7	6.6	36.3	2.9
469	C0/C17	C0/C17_DHL215	68.8	69.2	-0.5	193.9	91.6	18	38.1	11.8
470	C0/C17	C0/C17_DHL216	63.9	64.1	-0.4	164.8	63.4	36.8	37.2	10.8
471	C0/C17	C0/C17_DHL217	68.4	70.3	-1.6	180.2	93.7	35.3	40.1	8.5
472	C0/C17	C0/C17_DHL220	64.4	63.9	0.2	181.1	69.5	30.8	33.5	8.1
473	C0/C17	C0/C17_DHL221	65.9	64.9	0.9	182.4	86	35.3	38	10.9
474	C0/C17	C0/C17_DHL222	61.1	61.6	-0.5	139.6	61.3	33	36.1	15.4
475	C0/C17	C0/C17_DHL224	69.7	71.4	-1.7	178.4	90.2	26.6	36	12.3
476	C0/C17	C0/C17_DHL225	65.9	65.4	0.5	156.2	73.8	27	32	7.3

477	C0/C17	C0/C17_DHL226	66.5	67.3	-0.8	158.5	69.9	36.8	35.3	9.9
478	C0/C17	C0/C17_DHL228	64	66.6	-2.4	147.2	66.8	25.5	34.7	5.3
479	C0/C17	C0/C17_DHL230	66.2	66.9	-0.6	152.4	61	27	38.4	9.2
480	C0/C17	C0/C17_DHL231	63.9	64.1	-0.3	177.6	74.4	27	35.9	7.3
481	C0/C17	C0/C17_DHL232	63.8	63.5	0.1	163.9	85.1	45.1	41.5	10.1
482	C0/C17	C0/C17_DHL234	67.5	70.6	-2.9	144.4	70.3	27.8	33.2	11.6
483	C0/C17	C0/C17_DHL235	66.2	66.8	-0.4	192.3	102.6	23.3	41.4	15
484	C0/C17	C0/C17_DHL236	62	62.6	-0.5	176.7	71.7	20.3	39.1	7
485	C0/C17	C0/C17_DHL238	63.7	64.6	-0.9	178.2	77.9	27.8	40.5	10.3
486	C0/C17	C0/C17_DHL242	67	69.1	-1.9	159.6	85.2	33	29	10
487	C0/C17	C0/C17_DHL244	62.8	65.1	-2.2	169.6	71	27.8	36.9	9.3

MAFL - male flowering, FEFL - female flowering, ASI - anthesis–silking interval, PLHE - plant height, EAHE - ear height, FLA - flag leaf angle, TALE - tassel length, NPTB - number of primary tassel branches.

Supplementary Table 3. Significant SNP markers information based on the entire panel of DH lines. SNP markers with phenotypic variance explained (PEV) higher than 5% are highlighted in bold.

Trait	SNP	Chr	Position (bp)	P-value	MAF	Effect	PEV (%)
Female flowering	S2_212952913	2	212952913	8.53×10^{-8}	0.16	0.66	3.23
	S7_175855698	7	175855698	8.38×10^{-7}	0.12	0.72	4.75
	S9_7631219	9	7631219	2.69×10^{-6}	0.35	0.57	2.44
Flag leaf angle	**S1_199708767**	**1**	**199708767**	$\mathbf{1.86\times10^{-6}}$	**0.11**	**-3.43**	**9.30**
	**S2_168264788**	**2**	**168264788**	$\mathbf{2.57\times10^{-10}}$	**0.11**	**4.76**	**5.25**
	S7_34634474	7	34634474	2.39×10^{-8}	0.20	-3.23	1.19
	S7_40387690	7	40387690	2.43×10^{-9}	0.21	4.37	2.79
	S8_148977083	8	148977083	3.23×10^{-7}	0.22	3.28	1.31
	S9_19080430	9	19080430	8.41×10^{-8}	0.06	6.08	2.58
	S10_110663211	10	110663211	2.39×10^{-6}	0.34	-2.26	1.27
Number of primary tassel branch	S1_14846281	1	14846281	4.54×10^{-8}	0.16	-1.17	0.93
	S1_241800864	1	241800864	9.13×10^{-8}	0.26	-1.68	1.89
	S2_37816138	2	37816138	2.65×10^{-6}	0.45	0.76	0.44
	**S2_194820318**	**2**	**194820318**	$\mathbf{5.13\times10^{-9}}$	**0.14**	**1.82**	**5.13**
	S3_181430700	3	181430700	2.31×10^{-7}	0.05	-1.96	2.47
	S4_24421492	4	24421492	2.20×10^{-6}	0.08	-1.44	1.31
	S6_5181787	6	5181787	4.79×10^{-7}	0.49	-1.17	0.89
	S7_40387690	7	40387690	1.23×10^{-7}	0.21	1.59	1.45
	S7_172382655	7	172382655	2.06×10^{-8}	0.29	-1.04	1.14
	S7_181798181	7	181798181	9.27×10^{-10}	0.11	-1.85	2.96
	S8_136861313	8	136861313	3.56×10^{-9}	0.09	-1.80	1.94
	S9_114409650	9	114409650	2.51×10^{-7}	0.18	1.09	1.03

Supplementary Table 4. Allele frequencies (*p* and *q*) within population (C0_DHL, C17_DHL and C0C17_DHL) for the significant SNPs detected by GWAS using the entire panel.

Trait	SNP	C0_DHL		C17_DHL		C0C17_DHL	
		p	*q*	*p*	*q*	*p*	*q*
Female flowering	S2_212952913	0.62	0.38	1.00	0.00	0.84	0.16
	S7_175855698	0.70	0.30	1.00	0.00	0.88	0.12
	S9_7631219	0.22	0.78	0.97	0.03	0.63	0.37
Flag leaf angle	S1_199708767	0.75	0.25	1.00	0.00	0.86	0.14
	S2_168264788	0.67	0.33	1.00	0.00	0.91	0.09
	S7_34634474	0.92	0.08	0.71	0.29	0.79	0.21
	S7_40387690	0.34	0.66	0.99	0.01	0.91	0.09
	S8_148977083	0.47	0.53	1.00	0.00	0.75	0.25
	S9_19080430	0.92	0.08	1.00	0.00	0.95	0.05
	S10_110663211	0.64	0.36	0.69	0.31	0.62	0.38
Number of primary tassel branch	S1_14846281	0.79	0.21	0.85	0.15	0.87	0.13
	S1_241800864	0.26	0.74	1.00	0.00	0.82	0.18
	S2_37816138	0.64	0.36	0.48	0.52	0.54	0.46
	S2_194820318	0.70	0.30	1.00	0.00	0.82	0.18
	S3_181430700	0.82	0.18	1.00	0.00	0.97	0.03
	S4_24421492	0.76	0.24	0.99	0.01	0.95	0.05
	S6_5181787	0.08	0.92	0.99	0.01	0.32	0.68
	S7_40387690	0.34	0.66	0.99	0.01	0.91	0.09
	S7_172382655	0.47	0.53	0.99	0.01	0.56	0.44
	S7_181798181	0.61	0.39	1.00	0.00	0.96	0.04
	S8_136861313	0.87	0.13	1.00	0.00	0.84	0.16
	S9_114409650	0.79	0.21	0.89	0.11	0.75	0.25
